# Preclinical evaluation of 3D185, a novel potent inhibitor of FGFR1/2/3 and CSF-1R, in FGFR-dependent and macrophage-dominant cancer models

**DOI:** 10.1186/s13046-019-1357-y

**Published:** 2019-08-22

**Authors:** Xia Peng, Pengcong Hou, Yi Chen, Yang Dai, Yinchun Ji, Yanyan Shen, Yi Su, Bo Liu, Yueliang Wang, Deqiao Sun, Yuchen Jiang, Chuantao Zha, Zuoquan Xie, Jian Ding, Meiyu Geng, Jing Ai

**Affiliations:** 1 Division of Anti-Tumor Pharmacology, State Key Laboratory of Drug Research, Chinese Academy of Sciences, Shanghai Institute of Materia Medica, No. 555 Zuchongzhi Road, Pudong New District, Shanghai, 201203 People’s Republic of China; 2 University of Chinese Academy of Sciences, No.19(A) Yuquan Road, Shijingshan District, Beijing, 100049 People’s Republic of China; 3 Shanghai HaiHe Pharmaceutical Co., Ltd, No. 421 Newton Road, Zhangjiang Hi-Tech Park, Pudong New District, Shanghai, 201203 People’s Republic of China

**Keywords:** Kinase inhibitor, 3D185, FGFR, CSF-1R, Tumor microenvironment

## Abstract

**Background:**

The interaction between tumor cells and their immunosuppressive microenvironment promotes tumor progression and drug resistance. Thus, simultaneously targeting tumor cells and stromal cells is expected to have synergistic antitumor effects. Herein, we present for the first time a preclinical antitumor investigation of 3D185, a novel dual inhibitor targeting FGFRs, which are oncogenic drivers, and CSF-1R, which is the major survival factor for protumor macrophages.

**Methods:**

The antitumor characteristics of 3D185 were assessed by a range of assays, including kinase profiling, cell viability, cell migration, immunoblotting, CD8^+^ T cell suppression, and in vivo antitumor efficacy, followed by flow cytometric and immunohistochemical analyses of tumor-infiltrating immune cells and endothelial cells in nude mice and immune-competent mice.

**Results:**

3D185 significantly inhibited the kinase activity of FGFR1/2/3 and CSF-1R, with equal potency and high selectivity over other kinases. 3D185 suppressed FGFR signaling and tumor cell growth in FGFR-driven models both in vitro and in vivo. In addition, 3D185 could inhibit the survival and M2-like polarization of macrophages, reversing the immunosuppressive effect of macrophages on CD8^+^ T cells as well as CSF1-differentiated macrophage induced-FGFR3-aberrant cancer cell migration. Furthermore, 3D185 inhibited tumor growth via remodeling the tumor microenvironment in TAM-dominated tumor models.

**Conclusions:**

3D185 is a promising antitumor candidate drug that simultaneously targets tumor cells and their immunosuppressive microenvironment and has therapeutic potential due to synergistic effects. Our study provides a solid foundation for the investigation of 3D185 in cancer patients, particularly in patients with aberrant FGFR and abundant macrophages, who respond poorly to classic pan-FGFRi treatment.

**Electronic supplementary material:**

The online version of this article (10.1186/s13046-019-1357-y) contains supplementary material, which is available to authorized users.

## Background

In this era of personalized medicine, targeted therapies are used for specific cancer patients based on molecular alterations. Mutations in fibroblast growth factor receptors (FGFRs), including FGFR1, FGFR2, FGFR3, and FGFR4, are clinically relevant oncogenic drivers [1, 2]. Constitutive FGFR signaling is known to be involved in tumor cell proliferation and growth, angiogenesis and metastasis [3–7]. An investigation of 4853 solid tumors found that FGFR aberrations, including chromosomal translocation (8%), amplification (66%), and mutation (26%), are common in many cancers (7.1% of cancers). In addition, the most common FGFR-aberrant cancers are urothelial (32%), breast (18%), squamous cell non-small cell lung (13%), endometrial (13%), and ovarian (9%) cancers [8]. Moreover, activated FGFR signaling confers resistance to various anticancer therapies [9–11]. Taken together, these findings indicate that FGFR is a promising target for cancer treatment.

Numerous pharmaceutical companies and research institutes have been involved in the development of FGFR inhibitors [1, 5, 12]. Some of the FGFR inhibitors that have entered clinical trials showed promising clinical benefits and application potential [3, 13, 14]. However, many FGFR inhibitors under investigation are multitarget kinase inhibitors that are approved for kinase insert domain receptor (KDR)-targeted antiangiogenic therapy and significantly inhibit KDR and platelet-derived growth factor receptor (PDGFR) kinase activity, with much weaker activity against FGFR kinase [15–19]. The effects of these inhibitors against classical angiogenic kinases, especially KDR, may lead to severe hypertension and dose-limiting toxicity, which greatly impedes the ability of FGFR inhibitors to maximize the blockade of FGFR signaling at a relatively well-tolerated dose in patients harboring aberrant FGFR [20–22]. Thus, selective FGFR inhibitors, particular inhibitors with much weaker activity against KDR, are needed.

In the present study, we developed a novel FGFR inhibitor, 3D185 (also named HH185), that potently inhibited FGFR1/2/3 kinase activity, with over 760-fold selectivity for FGFR1 compared to KDR and other angiogenic kinases. Moreover, 3D185 significantly suppressed the activity of CSF-1R, with equal potency against FGFR1/2/3. The CSF-1/CSF-1R axis plays a key role in macrophage differentiation, proliferation and survival, especially for M2-like tumor-associated macrophages (TAMs) [23, 24]. Macrophages can account for up to 50% of leukocytes in the tumor microenvironment and are involved in various tumor-promoting processes that mediate tumorigenesis and drug resistance [25, 26], especially the evasion of immune surveillance and the suppression of CD8^+^ T cell expansion and function. Thus, targeting TAMs via CSF-1R inhibition is a promising approach for inducing antitumor effects and enhancing sensitivity to kinase-targeted therapy.

3D185, which simultaneously targets FGFR and CSF-1R, is expected to both inhibit tumor cells and remodel the tumor microenvironment to synergistically antagonize tumors and delay the development of resistance to FGFR inhibitors alone. Indeed, 3D185 inhibited FGFR signaling and tumor cell growth in representative aberrant FGFR-driven in vitro and in vivo models. The efficacy of 3D185 is comparable to that of the most advanced selective FGFR1/2/3 inhibitor, AZD4547. Moreover, 3D185 could inhibit the survival and M2-like polarization of macrophages, reversing the immunosuppressive effect of macrophages on CD8^+^ T cells. In the available in vivo TAM-dominated tumor models, oral administration of 3D185 remodeled the tumor microenvironment and delayed tumor growth. In addition, 3D185 inhibited CSF-1-differentiated macrophages induced FGFR3-aberrant cancer cell migration with potency much better than AZD4547 and PLX-3397. 3D185 was approved for investigational new drug [27] by the former China Food and Drug Administration (CFDA) (now renamed NMPA) in January 2018 and entered into phase I trials (www.chinadrugtrials.org.cn; study ID: CTR20181147). In the current study, we demonstrated for the first time the preclinical antitumor activity of 3D185 in FGFR-driven and CSF-1R-dependent models.

## Methods

### Compounds

**Fig. 1:**
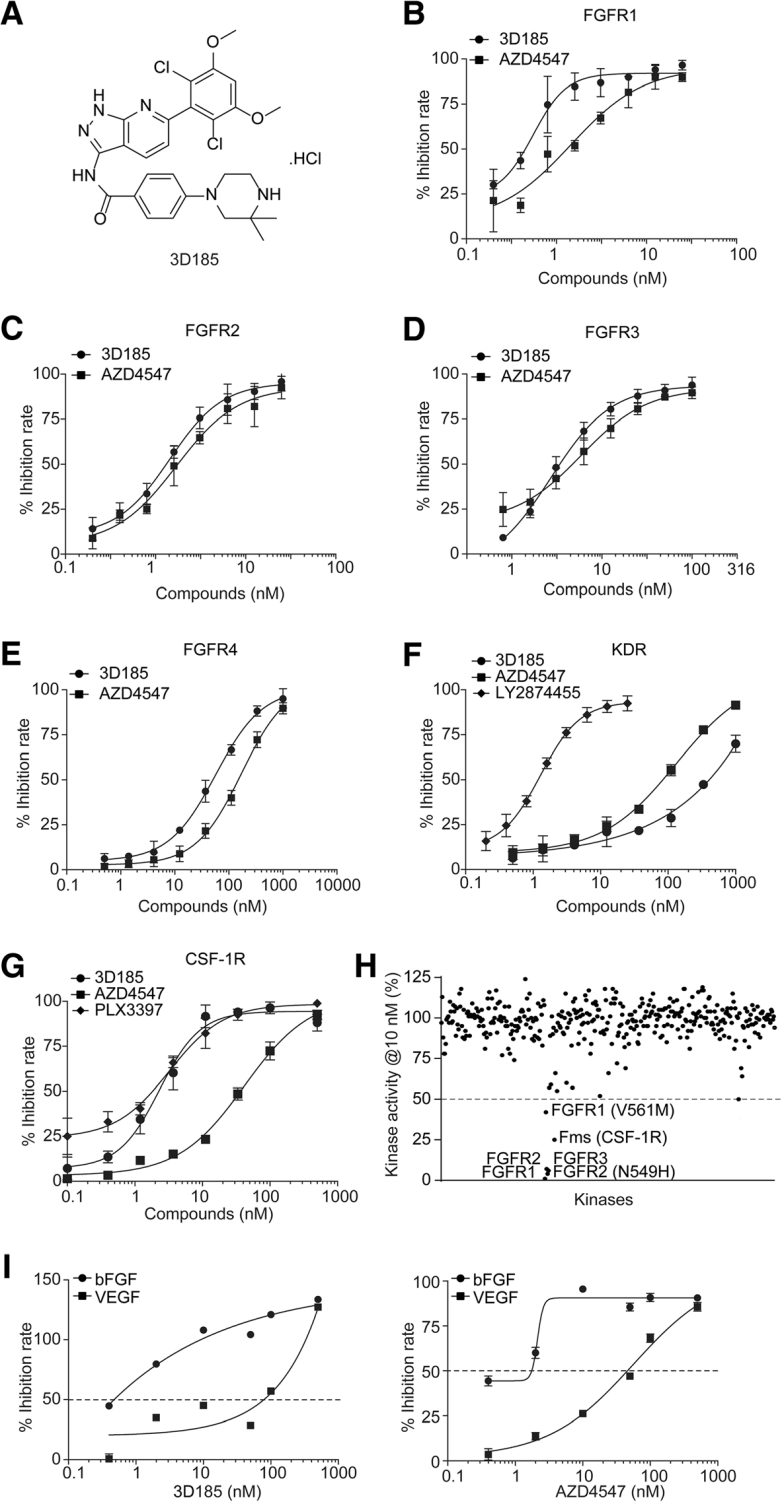
3D185 is a dual FGFR1/2/3 and CSF-1R inhibitor with high selectivity. **a** Chemical structure of 3D185. B-G, The kinase inhibition curves of 3D185 against FGFR1 (**b**), FGFR2 (**c**), FGFR3 (**d**), FGFR4 (**e**), KDR (**f**) and CSF-1R (**g**). H, A broad kinase profile of 3D185 against 372 protein kinases. I, The effect of 3D185 and AZD4547 on the cell viability of bFGF-induced or VEGF-induced HUVECs. Prestarved primary HUVECs were treated with bFGF or VEGF (100 ng/mL) and 3D185 or AZD4547 for 48 h, and cell viability was then measured using a CCK-8 assay. Representative data from three independent experiments are shown

3D185 (also named HH185, Fig. 1a) was synthesized at Shanghai HaiHe Pharmaceutical Co., Ltd. 3D185 was dissolved in dimethyl sulfoxide at a concentration of 10^− 2^ M and diluted to different concentrations for testing. AZD4547 and PLX3397 were purchased from Selleck Chemicals (Boston, United States).

### Cell culture

RT112, OPM2, CAL51, HCC366 and HCC78 cells were obtained from Deutsche Sammlung von Mikroorganismen und Zellkulturen GmbH (Braunschweig, Germany). EBC-1, MKN45, MKN1 and MKN28 cells were purchased from JCRB Cell Bank (Tokyo, Japan). UMUC14 cells were obtained from the European Collection of Cell Cultures (Salisbury, UK). SUM52PE cells were obtained from Asterand Company. MGC-823 and MDA-MB-453 cells were obtained from the Institute of Biochemistry and Cell Biology, Chinese Academy of Sciences (Shanghai, China). SGC-7901 cells were obtained from the Shanghai Cancer Institute, Renji Hospital, Shanghai Jiaotong University School of Medicine (Shanghai, China). Human umbilical vein endothelial cells (HUVECs) were obtained from ScienCell Research Laboratories (Shanghai, China). Other cell lines used in the present study were purchased from American Type Culture Collection (Manassas, United States). Some background information of cell lines used in the study was listed in Additional file 1: Table S1. All cell lines were obtained between 2000 and 2017 and cultured according to the provided instructions. The cells were confirmed to be free of mycoplasma and were passaged no more than 25–30 times after thawing. The cell lines were characterized using short tandem repeat markers by Genesky Biopharma Technology (most recent test in 2017).

### Antibodies and growth factors

Recombinant human bFGF and VEGF and mouse CSF-1 and CSF-2 were acquired from PeproTech Inc. (Rocky Hill, United States). Recombinant human CSF-1 and CSF-2 were acquired from R&D Systems (Minneapolis, United States). Antibodies specific for phospho-FGFR (Tyr653/654), FGFR1, FGFR2, FGFR3, phospho-CSF-1R (Tyr 723), CSF-1R, phospho-ERK (Thr202/Tyr204), ERK, phospho-PLCγ (Tyr783), PLCγ, phospho-Akt (Ser473), Akt and FoxP3 were purchased from Cell Signaling Technology (Danvers, United States); antibodies specific for phosphotyrosine (PY99) and CD31 were purchased from Santa Cruz Biotechnology (Dallas, United States); the antibody specific for Ki67 was purchased from Epitomics Inc. (San Francisco, United States); and the antibody specific for GAPDH was purchased from Kangcheng Bio (Shanghai, China).

### Kinase inhibition assay

Active FGFR1–4, VEGFR1, KDR, VEGFR3, PDGFRα and PDGFRβ proteins were purchased from Eurofins (Fremont, United States), and kinase activity was assessed using ELISA, as previously reported [28]. The broader kinase selectivity profile of 3D185 (0.01, 0.1, and 1 μM) was determined by Eurofins [29] by screening the compound against 372 human recombinant kinases. Increasing concentrations of ATP were diluted for the kinase reaction in the ATP competition assay. CSF-1R kinase activity was analyzed using the Z’-LYTE™ Kinase Assay Kit (Thermo Fisher, United States). The assay formats are described in the SelectScreen Kinase Profiling Service Zlyte protocol. IC_50_ values were calculated by dose-response curve fitting using Prism (Graph Pad).

### CD8^+^ T cell suppression assay

Spleen cells were isolated from C57BL/6 mice, followed by red blood cell (RBC) lysis. Human CD8^+^ T cells were isolated from human peripheral blood mononuclear cells (HemaCare), followed by human CD8^+^ T cell isolation kit (Miltenyi). Then, 1–2 × 10^6^ cells/well were stimulated with αCD3/αCD28 with or without CFSE/Celltrace and cocultured with 1 × 10^5^ CSF-1-induced bone marrow-derived macrophages (BMDMs) or CSF-1-induced human macrophages in 48-well plates for 72 h using RPMI 1640 with 50 mM 2-mercaptoethanol and 10 ng/mL IL-2. Then, the spleen cells or human CD8^+^ T cells were collected and analyzed for CD8^+^ T cell proliferation and functional marker expression by flow cytometry. Cell proliferation was determined by blank bead-stimulated fluorescence^low^ cells after 3 days of incubation. After 72 h, we treated the cells with eBioscience™ Cell Stimulation Cocktail (plus protein transport inhibitors) for 4 h, and cell activation was then determined by the level of TNF-α, IFN-γ and granzyme B expression in CD8^+^ T cells by flow cytometry.

### Tumor cell migration assays

RT112 cells suspended in 2% serum medium (1 × 10^5^ cells per well) were seeded in 24-well Transwell plates (pore size, 8 μm; Corning). The bottom chambers were filled with or without 0.5 × 10^5^ CSF-1-induced human macrophages, and appropriate controls or designated concentrations of compounds were added to both sides of the membrane. The cultures were maintained for further 24 h, and then the non-motile cells at the top of the filter were removed using a cotton swab. The migrating cells were fixed in paraformaldehyde (4%) and stained with crystal violet (0.1%) for 15 min at room temperature. The dye that was taken up by the cells bound to the membrane was released by the addition of 100 μL 10% acetic acid, and the absorbance of the resulting solution was measured at 595 nm using a multiwell spectrophotometer (SpetraMAX 190, from Molecular Devices, Palo Alto, CA, USA). The assay was performed in triplicate. Images were obtained using an Olympus BX51 microscope.

### Flow cytometry

For in vitro analysis, BMDMs or peripheral blood mononuclear cells (PBMCs) were collected, stained with a fluorescent surface marker antibody or the matching isotype controls for 30 min at room temperature and then tested using a BD LSRFortessa™. Data were analyzed using FlowJo software.

For analyses of tumor-infiltrating immune cells, MC38 mouse tumors were minced and digested using a Mouse Tumor Dissociation Kit (Miltenyi, Germany). The cells were passed through a 70 μm cell strainer, and single-cell suspensions were then analyzed by a BD LSRFortessa™, as described above. Antibodies specific for the following proteins and the matching isotype control or FMO control were used to analyze the leukocyte infiltrate: CD45, CD11b, F4/80, CD206, CD86, CSF-1R, CD3e, CD8a, CD25, CD4, IFN-γ, Ly-6C, Ly-6G and FoxP3(BD, eBioscience and Biolegend). Viability was determined by staining with either the LIVE/DEAD® Fixable Violet Dead Cell Stain Kit (Invitrogen) or the Zombie Aqua™ Fixable Viability Kit (Biolegend). The gating strategies for flow cytometry were showed in Additional file 1: Figure S1.

### In vivo antitumor activity assay

For the NCI-H1581 and SNU16 xenografts, female nude mice (4–6 weeks old) were housed and maintained under specific pathogen-free conditions. Tumor cells (5 × 10^6^ in 200 μL) were injected subcutaneously (s.c.) into the right flanks of nude mice and allowed to grow to 700–800 mm^3^, then cut into 1-mm^3^ fragments and transplanted s.c. into the right flanks of nude mice using a trocar. When the tumor volume reached 100–150 mm^3^, the mice were randomly assigned to the vehicle control group (n = 12) or a treatment group (n = 6 per group). The vehicle group received vehicle only, and the treatment groups received 3D185 or AZD4547 at the indicated doses via oral administration once daily for 14 (NCI-H1581) or 21 (SNU16) days.

For the MC38 xenograft model, 6-week-old C57BL/6 mice were housed and maintained under specific pathogen-free conditions. Tumor cells (2 × 10^6^ in 200 μL) were injected subcutaneously (s.c.) into the right flanks of C57BL/6 mice. When the tumor volume grew to 50–100 mm^3^, treatment of 8 mice per group was initiated with the vehicle control, 3D185 or PLX3397 via oral administration once daily for 23 days.

The tumors were measured twice per week using a microcaliper. Tumor volume (TV) was calculated as follows: TV = (length × width^2^)/2. In addition, the individual relative tumor volume (RTV) was calculated as follows: RTV = Vt / V_0_, where Vt is the volume on a particular day and V_0_ is the volume at the beginning of the treatment. The RTV is shown on the indicated days as the median ± SEM for the indicated groups of mice. Percent (%) inhibition (TGI) values were measured on the final day of the study for the drug-treated mice compared to the vehicle-treated mice and were calculated as 100 × {1 - [(V_Treated Final day_ - V_Treated Day 0_) / (V_Control Final day_ - V_Control Day 0_)].

Animal procedures were approved by the Institutional Animal Care and Use Committee of the Shanghai Institute of Materia Medica (approval No. 2016–04-DJ-21, 2017–02-GMY-04). According to our rules, the experiment should be terminated when the xenograft size reaches 3000 mm^3^, and the mice will be sacrificed. However, in our case, H1581 and SNU-16 tumors grow too fast. The compounds may not display their anti-tumor effects in such a short time. So we asked the veterinarian to reevaluate the animal condition and prolonged the experiment time. Overall, in our studies, tumor-bearing mice were still able to move, eat and drink freely, and in good condition.

### Statistical analyses

All statistical analyses were determined by one-way ANOVA with Tukey’s or Dunnett’s multiple-comparison test using GraphPad Prism 7.0 software (GraphPad).

## Results

### 3D185 Is a potent FGFR1/2/3 and CSF-1R inhibitor, with high selectivity against KDR

By performing medicinal chemistry-based studies, we discovered a potent inhibitor of FGFR1/2/3 kinase, namely, 3D185 (Fig. 1a). 3D185 exhibited potent inhibition of the kinase activity of FGFR1, FGFR2, and FGFR3, with IC_50_ values of 0.5, 1.3 and 3.6 nM, respectively (Additional file 1: Table S2, Fig. 1b-d), and exhibited much weaker activity against FGFR4 (IC_50_ = 51.4 nM) (Additional file 1: Table S2, Fig. 1e). The potency of 3D185 against FGFR1/2/3 kinase activity was comparable to that of AZD4547 (Additional file 1: Table S2, Fig. 1b-d), the most advanced selective FGFR inhibitor. The kinase selectivity of 3D185 was further screened in a large panel of 372 human kinases; 3D185 exhibited a much lower inhibitory effect against the activity of KDR kinase, with an IC_50_ of 381.5 nM (Additional file 1: Table S2, Fig. 1f). Moreover, the selectivity for FGFR1 over KDR was greater than 760-fold, which is much better than the selectivity of AZD4547 [30]. Excellent selectivity for FGFR was also observed over other typical angiogenesis-regulating kinases, including PDGFRα, PDGFRβ, VEGFR1 and VEGFR3 (> 2000-fold) (Additional file 1: Table S2).

Interestingly, 3D185 exhibited potent inhibition of the CSF-1R tyrosine kinase, and the IC_50_ value was 3.8 nM (Additional file 1: Table S2, Fig. 1g). Potency of 3D185 almost equal to that observed for FGFR1/2/3, suggesting that 3D185 may exert antitumor activity by simultaneously targeting the tumor microenvironment. This potency was almost comparable to that of PLX3397 (IC_50_ = 1.4 nM) (Additional file 1: Table S2, Fig. 1g), the most advanced CSF-1R inhibitor. Moreover, the activity of 3D185 against CSF-1R was much more potent than that of AZD4547 (IC_50_ = 40.3 nM) (Additional file 1: Table S2, Fig. 1g). However, consistent with the reported data [31], compared to the inhibitory activity of AZD4547 against FGFR, the potency of AZD4547 against CSF-1R was much weaker, which may limit the maximal therapeutic potential of on-target CSF-1R inhibition.

The broad kinase selectivity of 3D185 was investigated by screening an extended panel of 372 human recombinant protein kinases (including FGFR1–4 and CSF-1R). The data showed that 3D185 significantly inhibited the kinase activity of FGFR1/2/3, FGFR mutants (FGFR2 N549H and FGFR1 V561 M) and CSF-1R kinase, with IC_50_ values lower than 10 nM. In contrast, 3D185 had greater than 200- to 2000-fold (349 kinases) or 20-fold (17 kinases) selectivity for FGFR1 over the other 366 tested kinases (98.4%) (Fig. 1h, Additional file 1: Table S3), demonstrating the high selectivity of 3D185 for FGFR1/2/3 and CSF-1R. In addition, we chose FGFR1 as a representative kinase for kinetic studies. The results demonstrated that 3D185 functioned as an ATP-competitive inhibitor (Additional file 1: Figure S2A).

To further assess the selectivity of 3D185 for FGFR over KDR, bFGF- and VEGF-dependent proliferation assays were performed in primary HUVECs. 3D185 inhibited bFGF- and VEGF-induced primary HUVEC cell proliferation. The IC_50_ values were 0.5 and 88.1 nM (Fig. 1i, left panel), respectively. These findings indicated that 3D185 is much more potent (greater than 176-fold) in inhibiting HUVEC proliferation induced by bFGF than that induced by VEGF. Similarly, AZD4547 was also more potent in inhibiting HUVEC proliferation induced by bFGF than that induced by VEGF, but the fold change was only approximately 45-fold (Fig. 1i, right panel). The above results confirmed the higher selectivity of 3D185 than AZD4547 with respect to KDR. These results, together with the observation that 3D185 potently inhibited bFGF-induced phosphorylation of FGFR1, Erk and PLCγ (Additional file 1: Figure S2B, C), suggest that 3D185 potently inhibits FGFR activity with high selectivity over KDR.

Taken together, these results indicate that 3D185 is a selective FGFR1/2/3 and CSF-1R kinase inhibitor, which acts predominantly against FGFR1, FGFR2, FGFR3 and CSF-1R to antagonize tumors and the microenvironment simultaneously.

### 3D185 Blocks cellular FGFR signaling and CSF-1R signaling

**Fig. 2:**
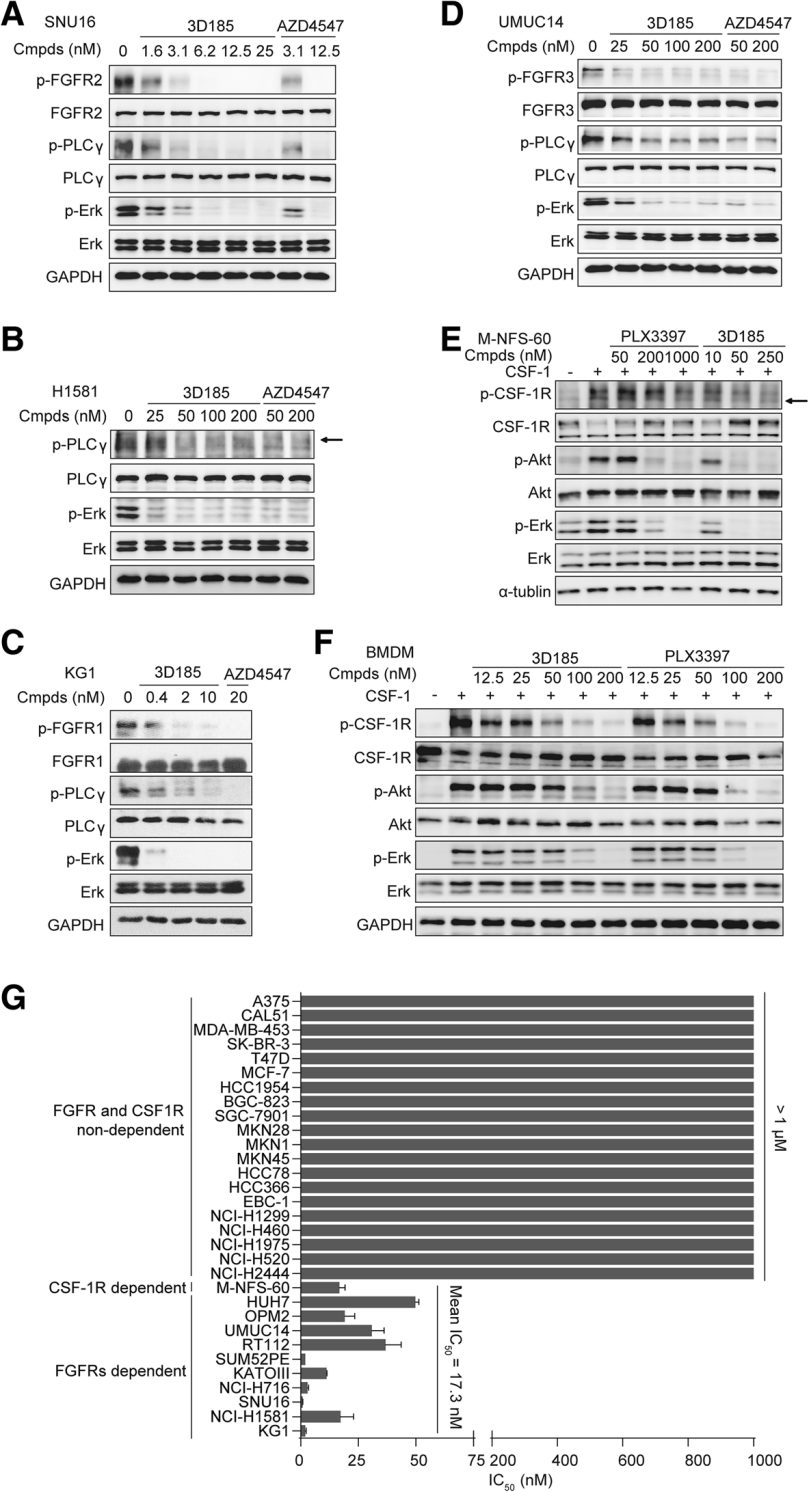
3D185 blocks cellular FGFR1/2/3 signaling and CSF-1R signaling, significantly inhibits FGFR-driven and CSF-1R-mediated cancer cell proliferation. A-D, SNU16 (**a**), NCI-H1581 (**b**), KG1 (**c**) and UMUC14 cells (**d**) were analyzed for total and phosphorylated levels of FGFR1/2/3, PLCγ and Erk. All cell lines were incubated for 2 h with 3D185, and the cells were then lysed and immunoblotted to detect the indicated proteins. E-F, M-NFS-60- (**e**) and CSF-1-differentiated mouse bone marrow-derived macrophages (BMDMs) (**f**) were analyzed for total and phosphorylated levels of CSF-1R, Akt and Erk. Both cell types were treated for 1 h with a series of concentrations of 3D185 and then incubated for 5 min with CSF-1. The cells were then lysed and immunoblotted to detect the indicated proteins. G, The antiproliferative activity of 3D185 was tested in a panel of cancer cell lines. The IC_50_ values are shown as the mean ± SD (nM) or as the estimated values from three independent tests

Furthermore, to evaluate the cellular effects of targeting FGFR1/2/3 and CSF-1R with 3D185, we chose the FGFR-driven and CSF-1R-dependent scenarios, respectively. First, four representative FGFR-aberrant cancer cell lines were used, namely, the KG-1 myeloid leukemia cancer cell line (FGFR1 translocation), NCI-H1581 lung cancer cell line (FGFR1 amplification), SNU16 gastric cancer cell line (FGFR2 amplification), and UMUC14 bladder cancer cell line (FGFR3 mutation). We analyzed p-FGFR and activation of its major downstream signaling molecules, p-Erk and p-PLCγ [3]. Phospho-FGFR1 is difficult to detect by immunoblotting in FGFR-amplified cancer cells because there is currently no reliable anti-phospho-FGFR1 antibody [30, 32]; therefore, we examined classic FGFR1 downstream signaling as a surrogate for FGFR1 activation in FGFR1-amplified NCI-H1581 cells. We found that 3D185 inhibited FGFR1/2/3 phosphorylation and Erk and PLCγ phosphorylation in a dose-dependent manner (Fig. 2a-d, Additional file 1: Figure S3A-D). Thus, at the cellular level, 3D185 potently inhibits phosphorylation of FGFR1, FGFR2, and FGFR3 and downstream signaling. In addition, the potency of 3D185 for the inhibition of FGFR1/2/3 signaling is comparable to that of AZD4547.

We also conducted a cellular phosphorylation assay of CSF-1R and the downstream signaling molecules Erk and Akt using a CSF-1-dependent murine myelogenous leukemia cell line (M-NFS-60) and CSF-1-differentiated mouse BMDMs treated with 3D185 or PLX3397. As reported previously, CSF-1-induced murine macrophages are thought to exhibit the M2-like polarization state of macrophages, suppressing T cell expansion and promoting tumor growth [24]. As expected, CSF-1R is overactive in M-NFS-60 and CSF-1-differentiated BMDMs. 3D185 significantly suppressed CSF-1R phosphorylation and downstream signaling (Fig. 2e, f, Additional file 1: Figure S3E, F). The efficacy of 3D185 was comparable to that of PLX3397. Taken together, these results showed that 3D185 not only blocks FGFR activation but also exhibits potent inhibitory activity against CSF-1R kinase.

### 3D185 Significantly inhibits FGFR-driven and CSF-1R-mediated cancer cell proliferation

Next, the impact of 3D185 on FGFR- and CSF-1R-mediated cancer cell proliferation was explored, and 11 FGFR- or CSF-1R-dependent cancer cell lines were chosen (Additional file 1: Table S4). As shown in Fig. 2g and Additional file 1: Table S4, 3D185 inhibited FGFR1-, FGFR2-, FGFR3-, and CSF-1R-mediated cancer cell proliferation, with IC_50_ values ranging from 0.9 to 36.8 nM. The concentration of 3D185 required to inhibit cellular FGFR signaling or CSF-1R signaling was consistent with the IC_50_ value for in vitro proliferation (Additional file 1: Table S4, Figs. 2a-f), indicating that 3D185 inhibits the proliferation of the above cancer cell lines via targeting FGFR or CSF-1R signaling. Moreover, the antiproliferative activity of 3D185 in FGFR-driven contexts is comparable to that of AZD4547. The potency of 3D185 in the CSF-1R-aberrant activated scenario is much higher than that of AZD4547 and comparable to that of PLX3397.

In contrast, 3D185 was inactive against 20 additional tumor cell lines that exhibited low expression or activation of FGFR and CSF-1R (IC_50_ > 1 μM) (Fig. 2g, Additional file 1: Table S5), further demonstrating that 3D185 is a highly potent and selective FGFR1/2/3 and CSF-1R inhibitor.

### 3D185 Inhibits CSF-1/CSF-1R-mediated survival and polarization of M2-like ‘protumor’ macrophages

The CSF-1/CSF-1R axis is essential for the differentiation and survival of M2-like TAMs [33]. We next investigated the effect of 3D185 on macrophage differentiation and survival after CSF-1 stimulation. Human macrophages were differentiated from monocytes by CSF-1 stimulation for 7 days [24]. We also used the granulocyte macrophage (GM)-CSF-stimulated context as a reference. CSF-1 induced M2-like ‘protumor’ TAMs, while GM-CSF regulated ‘antitumor’ Mo-DCs/macrophages [24, 34]. Similarly, murine macrophages were derived from bone marrow cultured in vitro with recombinant mouse CSF-1 for 7 days [35].

**Fig. 3:**
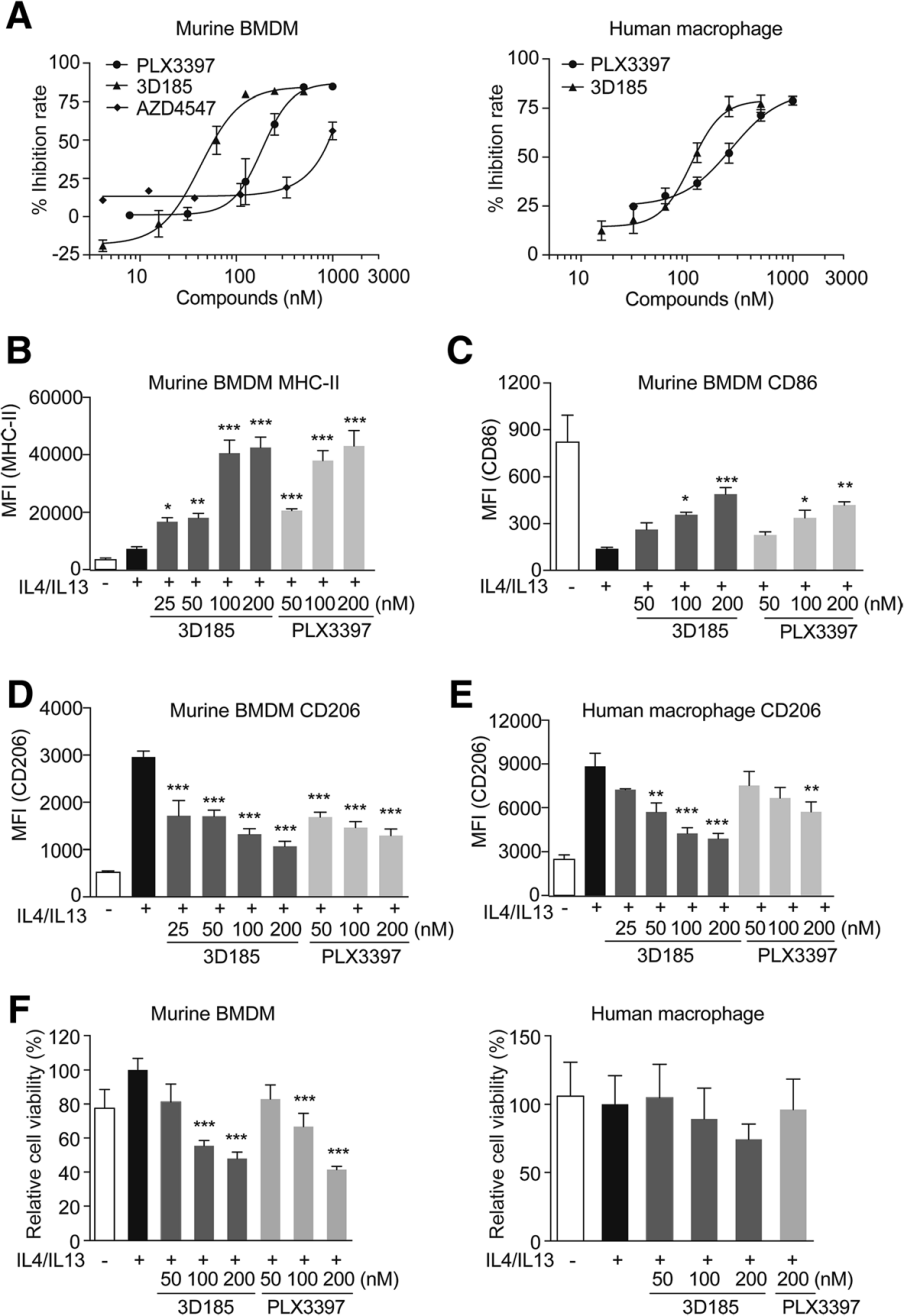
3D185 inhibited the survival and polarization of M2-like macrophages induced by CSF-1/CSF-1R. **a** The effect of 3D185 and PLX3397 on the survival of CSF-1-induced murine and human macrophages. Murine bone marrow and human monocytes were stimulated with CSF-1 for 7 days together with the indicated inhibitor, and cell viability was evaluated by the CCK-8 assay. The IC_50_ values are shown as the mean ± SD (nM) from two independent tests. B-E, To test the impact of 3D185 on the polarization of CSF-1-induced macrophages, murine bone marrow cells and human monocytes were induced to mature into macrophages with CSF-1 for 7 days and then stimulated with CSF-1, IL-4 and IL-13 for an additional 48 h. The expression of the markers MHC-II (**b**), CD86 (**c**), and CD206 (**d**) on induced murine macrophages and CD206 expression on induced human macrophages (**e**) were determined by flow cytometry, and the mean fluorescence intensity (MFI) was analyzed with FlowJo. Representative data from two independent experiments. **f** Murine bone marrow cells (left panel) and human monocytes (right panel) were induced to mature into macrophages with CSF-1 for 7 days and then stimulated with CSF-1, IL-4 and IL-13 and treated with vehicle or the indicated inhibitor for an additional 48 h. Then, all cells were collected to count viable cells by staining with trypan blue

As shown in Fig. 3a and Additional file 1: Table S6, 3D185 reduced the survival of CSF-1-induced murine and human macrophages, with IC_50_ values of 57.8 nM and 118.5 nM, respectively. The activity of 3D185 was much more potent than that of AZD4547 and comparable to that of PLX3397. In contrast, 3D185 and PLX3397 had no influence on the survival of GM-CSF-induced murine and human ‘antitumor’ Mo-DCs/macrophages, even at a concentration of 1 μM (Additional file 1: Table S6). Thus, 3D185 significantly inhibited the survival of CSF-1-induced M2-like ‘protumor’ macrophages.

Next, we tested whether 3D185 could also inhibit M2-like polarization of macrophages via inhibition of the CSF-1/CSF-1R axis. CSF-1-differentiated mouse BMDMs and CSF-1-differentiated human monocytes-derived macrophages were further treated with CSF-1, IL-4 and IL-13 for 48 h. CD206, a representative M2-type marker for macrophages, and CD86 and MHC-II, representative M1-type markers for macrophages, were examined by flow cytometric analysis. As expected, treatment with 3D185 inhibited the M2-like polarization of macrophages in both murine and human CSF-1-induced macrophages, as indicated by decreased CD206 expression and increased MHC-II and CD86 expression, in a dose-dependent manner (Fig. 3b-e). Consistent with this finding, 3D185 suppressed CSF-1R phosphorylation on macrophages costimulated with CSF-1, IL-4 and IL-13 (Additional file 1: Figure S4A). In addition, marginal inhibition of cell survival was observed (Fig. 3Ff) under conditions with significant inhibition of M2-like polarization of macrophages (Fig. 3b-e), indicating that inhibition of the survival of M2-like macrophages partially contributed to the inhibition of the M2-like polarization phenotype after 3D185 treatment.

Taken together, these results indicated that 3D185 was capable of reversing the M2-like polarization and survival of macrophages, and its potency was comparable to that of PLX3397.

### 3D185 Reversed M2-like macrophage-induced CD8^+^ T cell suppression

**Fig. 4:**
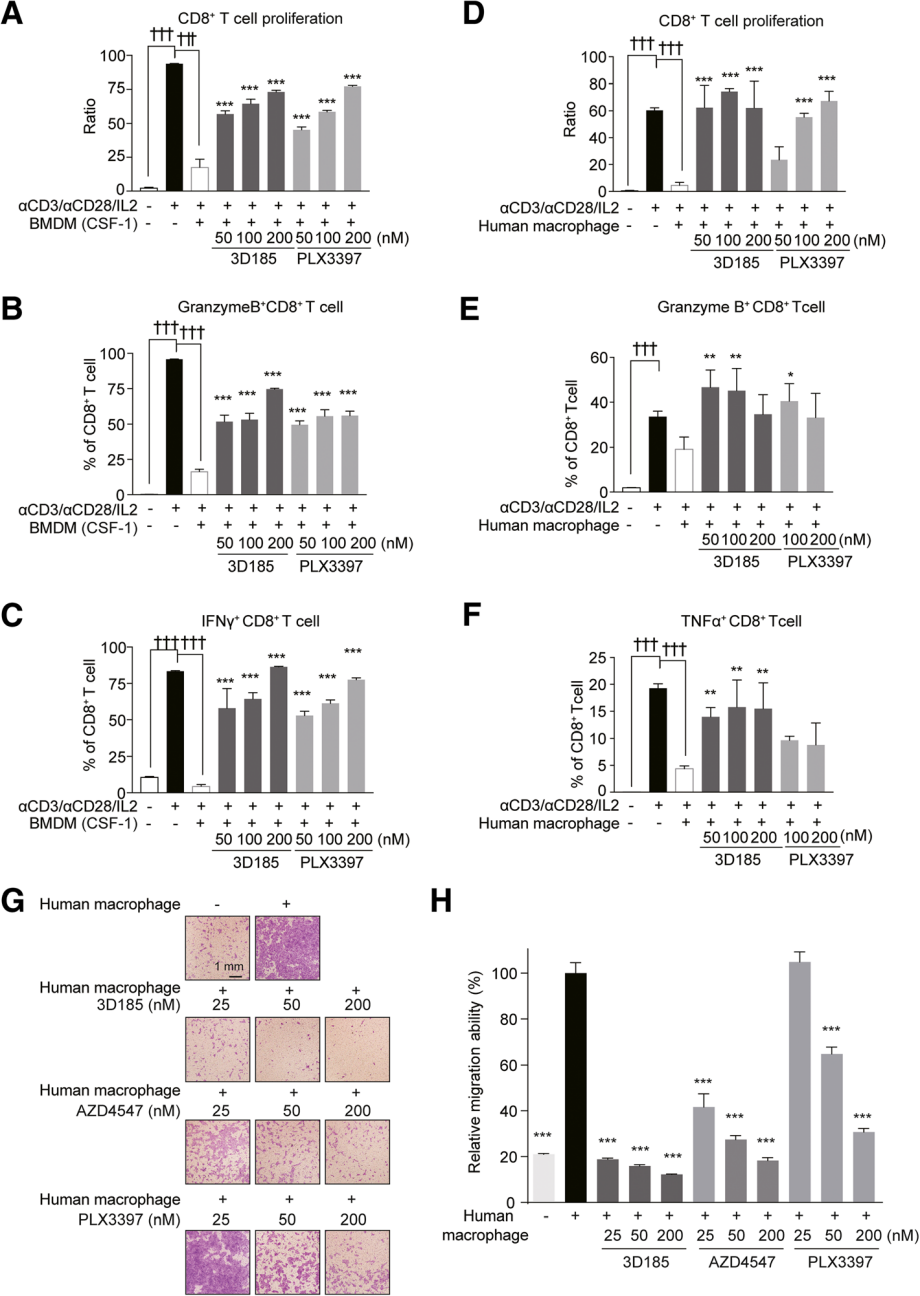
3D185 reversed M2-like macrophage-induced CD8^+^ T cell suppression as well as inhibited CSF-1-differentiated macrophages induced FGFR-aberrant cancer cell migration. **a-c** 3D185 reversed murine CSF1-differentiated macrophage-induced CD8^+^ T cell suppression. Murine bone marrow cells were induced to mature M2-like macrophages with CSF-1 for 7 days and then treated with 3D185 or PLX3397 for 48 h. Then, murine BMDMs were cocultured with CFSE-labeled spleen cells and stimulated with anti-CD3/CD28 beads and IL-2 for 72 h. CD8^+^ T cell proliferation was tested by flow cytometry (**a**). granzyme B^+^ CD8^+^ T cells (**b**) and IFN-γ^+^ CD8^+^ T cells (**c**) were tested by flow cytometry. Representative data from two independent experiments are shown. Data are shown as the mean ± SD. D-F, 3D185 reversed human CSF1-differentiated macrophage-induced CD8^+^ T cell suppression. Human monocytes cells were induced to M2-like macrophages with CSF-1 for 7 days and then treated with 3D185 or PLX3397 for 48 h. Then, such human macrophages were cocultured with CFSE-labeled human CD8^+^T cells and stimulated with anti-CD3/CD28 beads and IL-2 for 72 h. CD8^+^ T cell proliferation was tested by flow cytometry (**d**). granzyme B+ CD8^+^ T cells (**e**) and TNF-α^+^ CD8^+^ T cells (**f**) were tested by flow cytometry. Representative data from two independent experiments are shown. Data are shown as the mean ± SD. **g, h** CSF-1-differentiated macrophages induced FGFR3-amplified RT112 cancer cell migration. Representative images are shown (scale bars, 1 mm) in (**g**). The relative migration was plotted (**h**). The data shown are the mean ± SD from two independent experiments, assuming 100% migration or invasion of cells stimulated with macrophage. Significant differences were determined using one-way ANOVA with Tukey’s multiple-comparison test (*p < 0.05; **p < 0.01; ***p < 0.001)

M2-like macrophages can suppress T cell expansion and activation; thus, we further evaluated whether 3D185 could alleviate this impact by using murine macrophages as representative cells. As expected, when cocultured with autologous spleen cells, CSF-1-induced macrophages potently suppressed CD8^+^ T cell proliferation and decreased the ratio of activated CD8^+^ T cells. However, as expected, pretreatment of CSF-1-differentiated macrophages with 3D185 dramatically restored T cell proliferation and increased the ratio of activated CD8^+^ T cells, as evidenced by the increased ratio of IFN-γ and granzyme B expression in single- or double-positive CD8^+^ T cells (Fig. 4a-c, Additional file 1: Figure S4B-E). Consistently, similar results were also obtained on CSF-1-differentiated human monocytes-derived macrophages cocultured with CD8^+^ T cells, as evidenced by significant restored T cell proliferation and the increased ratio of activated CD8^+^ T cells upon 3D185 treatment (Fig. 4d-f, Additional file 1: Figure S4F-H). In addition, we observed similar effects in autologous spleen cells cocultured with CSF-1-differentiated M2-like BMDMs costimulated with CSF-1/IL-4/IL-13 after 3D185 treatment (data not shown). Thus, 3D185 can efficiently eliminate the immunosuppressive effect of M2-like macrophages.

### 3D185 Inhibited CSF-1-differentiated macrophages induced FGFR3-aberrant cancer cell migration with potency much better than AZD4547 and PLX-3397

From the above in vitro scenarios dominant by FGFR per se or CSF1/CSF1R axis per se, 3D185 exhibited equal potency with AZD4547 or PLX3397, respectively. To evaluate 3D185’ potential advantage of dual targeting FGFR and CSF1R with equal potency, we further used a co-cultured context with FGFR3-driven RT112 cancer cell line and CSF-1-differentiated human macrophages. We found that 3D185 strongly inhibited CSF-1-differentiated macrophages induced RT112 cell migration, blocking most cell migration at the dose of 25 nM. However, at the same dose, AZD4547 showed much weaker inhibitory effect and PLX3397 showed no inhibitory effect. All these suggested due to its dual targeting FGFRs and CSF-1R with equal potency, 3D185 could obtain a better efficacy in the context harboring FGFR alteration and rich of TAM in comparison to AZD4547 or PLX3397 (Fig. 4g-h).

### 3D185 Strongly inhibits FGFR-driven tumor growth in vivo at well-tolerated doses

**Fig. 5:**
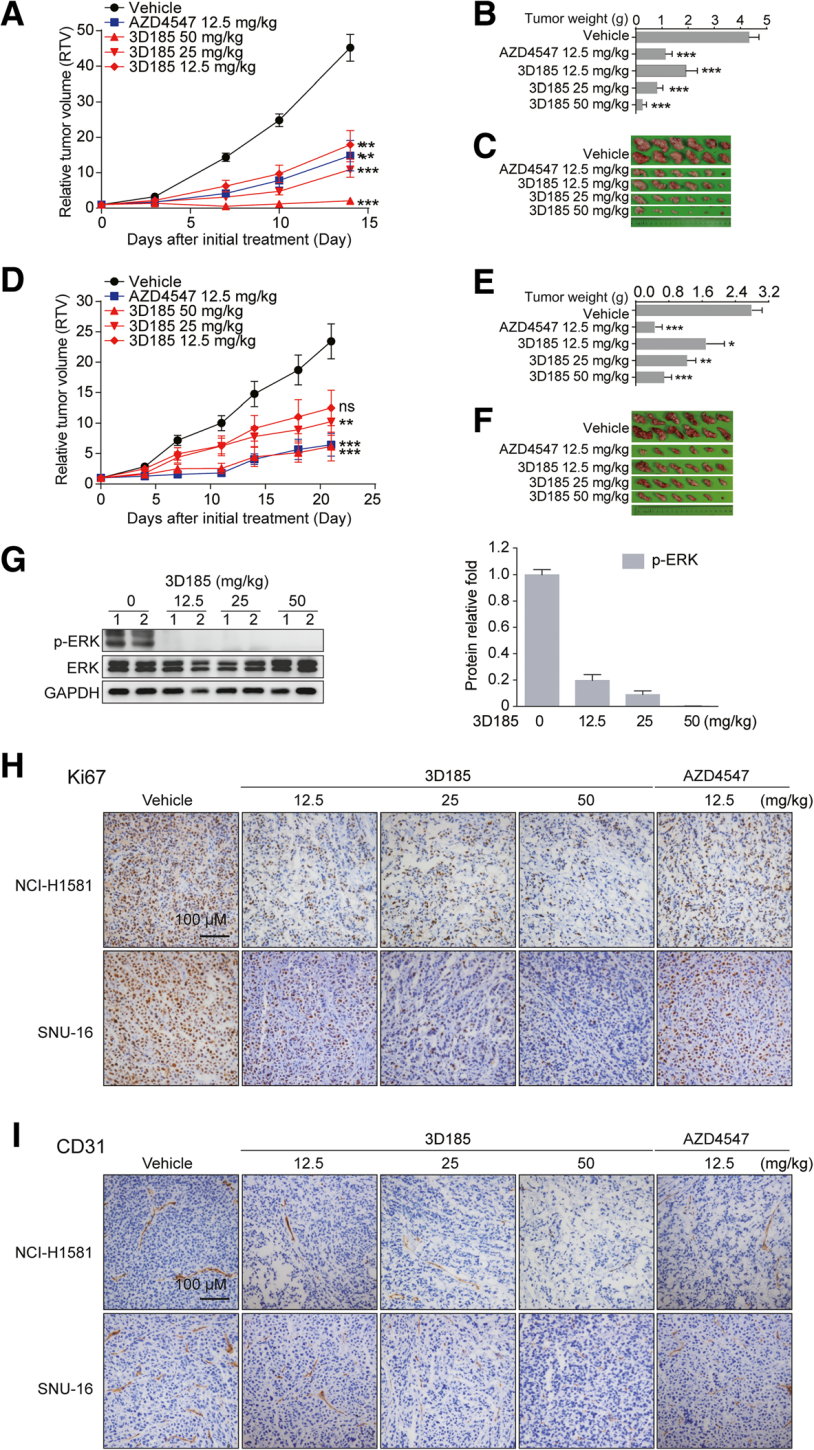
3D185 strongly inhibited FGFR-driven tumor growth in vivo. A-F, Antitumor efficacy of 3D185 in NCI-H1581 (**a-c**) and SNU16 (**d-f**) xenografts. Tumor growth inhibition after treatment with 3D185 is shown. The RTVs are presented as the mean ± SEM (**a, d**). Tumor weights (g) (**b, e**) and images of isolated subcutaneous xenograft tumors (**c, f**) are shown on the day after mice completed the 3D185 treatment course. *p < 0.05; **p < 0.01; ***p < 0.001, as determined by one-way ANOVA with Tukey’s multiple-comparison test. **g** 3D185 inhibited FGFR signaling in vivo. The intensity of phosphorylated protein band was quantified and normalized with the corresponding internal control protein band (right panel). Mice bearing NCI-H1581 subcutaneous tumor xenografts received a single oral dose of 3D185. Tumors were harvested at 6 h after treatment, and intratumoral p-ERK levels were tested by immunoblotting. **h-i** IHC evaluation of Ki67 (**h**) and CD31 (**i**) expression was performed in NCI-H1581 and SNU16 xenograft models after the final dose of 3D185 (scale bars, 100 μm)

Next, we investigate the in vivo antitumor effect of 3D185. The FGFR1-amplified NCI-H1581 cancer cell line was first used to create a subcutaneous xenograft model in nude mice. The results showed that 3D185 potently suppressed tumor growth. The tumor growth inhibition rates for 12.5, 25 and 50 mg/kg 3D185 were 60.4,74.9 and 96.4%, respectively (Fig. 5a-c). The efficacy of 12.5 mg/kg 3D185 was comparable to that of 12.5 mg/kg AZD4547. Additionally, 3D185 was well tolerated, as indicated by the absence of body weight loss in any treated group (Additional file 1: Figure S5A). Similar results were observed in the 3D185-treated FGFR2-amplified SNU16 model (Figs. 5d-f, Additional file 1: Figure S5B). These results indicate that 3D185 has robust antitumor efficacy at well-tolerated doses in FGFR-dependent tumor models. Consistent with the observed tumor growth suppression, there was a significant reduction in intratumoral FGFR signaling, as indicated by the nearly complete inhibition of ERK phosphorylation, at 6 h after administration of a single dose of 12.5, 25, or 50 mg/kg 3D185 in mice bearing NCI-H1581 tumors (Fig. 5g). These findings suggest that the antitumor efficacy of 3D185 can be attributed to FGFR-targeted inhibition.

To confirm the mechanism by which 3D185 inhibits tumor growth in vivo, NCI-H1581 and SNU16 tumors were processed for analysis of Ki67 and CD31, which are markers of cellular proliferation and vascularity, respectively. A significant dose-dependent decrease in Ki67 levels was observed after treatment with 3D185 (Fig. 5h, Additional file 1: Figure S5C). Similarly, after treatment with 3D185, a remarkable reduction in CD31-positive endothelial cells was observed (Fig. 5i, Additional file 1: Figure S5D). Taken together, these data show that the in vivo antitumor effect of 3D185 involves FGFR signaling inhibition, resulting from both antiproliferative and antiangiogenic effects.

### 3D185-induced CSF-1R inhibition results in remodeling of the tumor microenvironment and delayed tumor growth in a murine tumor model

**Fig. 6:**
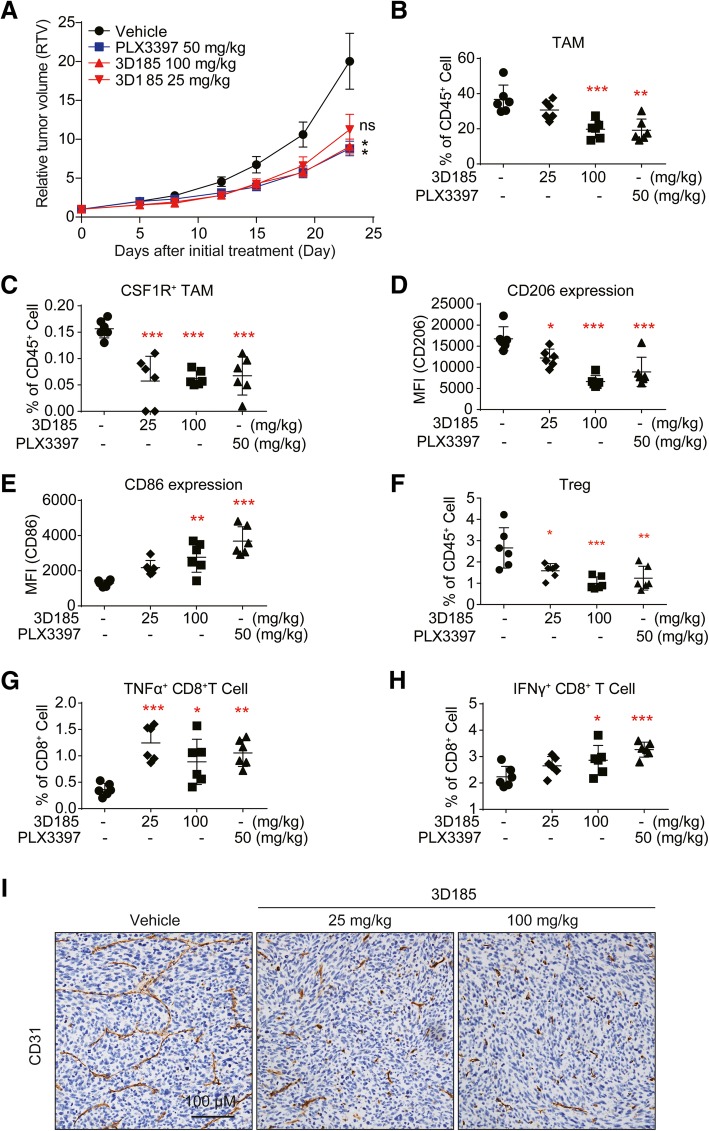
3D185 significantly inhibited MC38 tumor growth in vivo and remodeled the tumor microenvironment. **a** Antitumor efficacy of 3D185 in MC38 models. Tumor growth inhibition of the treatment with 3D185 is shown. The RTVs are presented as the mean ± SEM. *p < 0.05; **p < 0.01; ***p < 0.001, as determined by one-way ANOVA with Tukey’s multiple-comparison test. **b**-**g** Flow cytometric analysis of immune subsets in the MC38 tumor model treated with vehicle or the indicated inhibitor. Tumor tissues collected 2 h after treatment with 3D185. The infiltration of TAMs (**b**), CSF-1R^+^ TAMs (**c**), Treg (**f**),TNF-α^+^ CD8^+^ T cells (**g**) and IFN-γ^+^ CD8^+^ T cells (**h**) and the expression of CD206 (**d**) and CD86 (**e**) on TAMs were tested (n = 6 mice per group). Representative data from three independent experiments are shown. Data are shown as the mean ± SD. *p < 0.05; **p < 0.01; ***p < 0.001 vs the vehicle group, determined by one-way ANOVA with Dunnett’s multiple-comparison test. **i** IHC evaluation of CD31expression was performed on tumors from the MC38 tumor model after treatment with 3D185 (scale bar, 100 μm)

Next, we tested the efficacy of 3D185-inhibited CSF-1R activation in vivo. We used a murine MC38 colorectal adenocarcinoma model in immune-competent mice to represent TAM-dominant tumor models [24]. Both 3D185 and PLX3397 caused delayed tumor growth with equal potency (Fig. 6a). Significant body weight loss was not observed in any treated group (Additional file 1: Figure S5E). Next, we investigated whether CSF-1R targeting via 3D185 also influenced TAM turnover and infiltration of other immune cell subpopulations, especially CD8^+^ T cells, in vivo. MC38 tumor tissues were analyzed by flow cytometry and immunohistochemistry. As expected, following the administration of 3D185, we found markedly reduced infiltration of TAMs and CSF-1R^+^ TAMs (Fig. 6b, c). A significant reduction in TAMs was accompanied by a positive shift from ‘protumor’ M2-like TAMs to ‘antitumor’ M1-like TAMs upon treatment with 3D185 or PLX3397 (Fig. 6d, e). Notably, treatment with 3D185 also resulted in a decrease in T regulatory cells (Fig. 6f), the tumor-promoting subpopulation of CD4^+^ T cells, and a significant increase in the infiltration of activated CD8^+^ T cells (IFN-γ^+^ CD8^+^ T cells, TNF-α^+^ CD8^+^ T cells), though it did not affect the CD8^+^ T cells infiltration ((Fig. 6g, h, Additional file 1: Figure S5F). These results suggest that in addition to the substantial reduction of infiltrated TAMs, other immune cell subtypes may also be influenced by CSF-1R inhibition. In addition, given that TAMs promote angiogenesis during tumor progression, CD31 was examined in MC38 tumor sections. We found that CD31-positive endothelial cells were significantly reduced upon 3D185 treatment (Fig. 6i, Additional file 1: Figure S5G). All these findings using a murine MC38 model demonstrated that 3D185 could elicit antitumor efficacy by reversing an immunosuppressive tumor microenvironment.

## Discussion

Many advances in cancer treatment have been made through the development of oncogenic kinase-targeted therapy. The incorporation of tumor genotyping has led to remarkable responses in selected patients treated with matched TKIs [36]. Interestingly, in recent years, the tumor microenvironment has emerged as a target for the development of anticancer therapy since stromal tumor-promoting immune cells can sustain tumor growth, metastasis, and drug resistance [37–39]. Indeed, targeting the tumor-promoting microenvironment with immunotherapy has profoundly shaped the traditional approach to cancer treatment and has shown great therapeutic efficacy after application in many types of tumors. Among stromal cells, TAMs function as essential regulators of the complex tumor microenvironment, promoting tumor growth, angiogenesis, and metastasis [26, 40]. Furthermore, TAMs can exhibit potent suppression of cytotoxic T lymphocytes (CTLs). In contrast, depletion of TAMs can elicit antitumor responses by CD8^+^ T cells [40]. It is well known that the macrophage subtype with protumor effects is M2-like macrophages, in contrast to the antitumor M1-like subtype [24, 41, 42]. Thus, dual targeting of tumor cells and their local environment may have synergistic antitumor effects and delay the development of drug resistance.

Here, we developed a novel kinase inhibitor, 3D185, that targets FGFR1/2/3, which are clinically relevant oncogenic drivers, and CSF-1R, which is a crucial functional axis for immunosuppressive M2-like macrophages. Therefore, 3D185 has the ability to synergistically antagonize tumors via both tumor cells and TAMs.

Using FGFR-dependent cancer cell lines, we found that 3D185 potently inhibited FGFR1/2/3 signaling and, accordingly, significantly inhibited FGFR-mediated cancer cell proliferation and HUVEC survival. Consistent with the antiproliferative effects of 3D185 in five representative FGFR-aberrant cells, FGFR signaling was inhibited with a similar potency. In addition, using two representative FGFR-driven in vivo models, NCI-H1581 and SNU16, we showed that daily oral administration of 3D185 substantially inhibited tumor growth at well-tolerated doses. The efficacy of 3D185 in vitro and in vivo was comparable to that of AZD4547, the most advanced FGFR1/2/3 inhibitor.

In parallel, 3D185 could potently inhibit the proliferation of CSF-1/CSF-1R-dependent cancer cell lines and the survival of CSF-1-induced M2-like ‘protumor’ macrophages. Similarly, 3D185 reversed the M2-like polarization of TAMs. As such, this compound alleviated M2-like macrophage-induced CD8^+^ T cell suppression. In vivo, using the TAM-dominant murine MC38 cancer model, a dramatic reduction in tumor-infiltrating M2-like TAMs was observed, and other immune cell subpopulations were also affected by 3D185, including a decrease in FoxP3^+^ T regulatory cells and an increase in CD8^+^ T cells, especially activated CD8^+^ T cells. These results demonstrated the ability of 3D185 to combat the immunosuppressive tumor microenvironment.

However, the efficacy of 3D185 and another CSF-1R inhibitor, PLX3397, in the murine MC38 model was moderate, which is consistent with published data [24, 43]. Preclinical studies using mouse models observed that CSF1R blockade dramatically reduced TAMs but accompanied by an accumulation of tumor-infiltrating bone marrow-derived suppressor cells (MDSCs), which may result in moderate efficacy for CSF-1R inhibitors [40, 44–46]. Consistent with this finding, we also detected increased infiltration of MDSCs upon treatment with 3D185 and PLX3397 (Additional file 1: Figure S5H). Moreover, CSF1R inhibitor combined with the treatment abrogating the increased MDSC infiltration resulted in significant delay in tumor progression [44]. Therefore, further clinical investigation of this combination strategy is promising.

As mentioned previously, many FGFR inhibitors under clinical investigation are multitargeted kinase inhibitors that retain high KDR inhibitory activity. Although in both preclinical and clinical studies, antiangiogenic tumor therapy showed a survival benefit, this approach induced serious side effects that limited the duration of exposure in cancer patients. In addition, some preclinical investigations have indicated that such treatment may have the risk of accelerating metastasis. In contrast, our compound acted predominantly against FGFR1/2/3 and CSF-1R, with considerably less activity against KDR and other classic angiogenic kinases, which was confirmed by a molecular kinase assay (372 kinds) and cellular bFGF- and VEGF-induced HUVEC proliferation. Moreover, 3D185 was more selective than AZD4547 for FGFR over KDR (molecular assays: 64.4-fold; cellular assays: 45-fold). Thus, 3D185 could maximize the therapeutic potential against the targets FGFR and CSF-1R in cancer patients and avoid the classic toxic effects associated with KDR. It is worth noting that although clinical progress has been made in FGFR-aberrant bladder cancer and intrahepatic cholangiocarcinoma treated with pan-FGFR inhibitors, FGFR-aberrant cancers, such as squamous cell lung cancer and breast cancer, which have an abundance of macrophages, have shown poor responses to FGFRi treatment alone [47–50]. Indeed, our recent published work suggested that macrophages could interact with cancer cells to enhance resistance to FGFR inhibitors [51]. Moreover, in a co-cultured context with FGFR3-driven cancer cell line and CSF-1-differentiated human macrophages, 3D185 inhibited macrophages induced cancer cell migration with potency much better than AZD4547 and PLX-3397. Therefore, based on its dual inhibition of FGFRs and CSF-1R with equal potency, 3D185 may improve clinical efficacy in tumors with FGFR aberrance and abundant of macrophages.

## Conclusions

3D185 is a dual inhibitor against FGFR1/2/3 and CSF-1R that exhibits potent antitumor activities. This compound is promising because it simultaneously targets tumor cells per se and the immunosuppressive tumor microenvironment to synergistically antagonize tumors. Based on these advantages, 3D185 is now in phase I clinical trials.

## Additional file

Additional file 1: **Table S1.** Some background information of cell lines used in the study. **Table S2.** Enzymatic kinase activity of the 3D185 and AZD4547. **Table S3.** Profiling of 3D185 against 372 kinases by Eurofins. **Table S4.** Anti-proliferative activity of 3D185 in sensitive cell lines. **Table S5.** Antiproliferative assay used in indicated cell lines in Fig. 2. **Table S6.** The effect of 3D185 and PLX3397 on cell survival of CSF-1-differentiated or GM-CSF-differentiated murine and human ‘protumor’ macrophages or ‘antitumor’ Mo-DCs/macrophages. **Figure S1.** The gating strategies for Macrophage, CD8^+^ T cell, T_reg_ in flow cytometry analyses of tumor-infiltrating immune cells. **Figure S2.** 3D185 inhibited FGFR1 kinase activity in an ATP-competitive manner and inhibited bFGF-stimulated FGFR signaling in primary HUVECs. **Figure S3.** The intensity of phosphorylated protein band was quantified and normalized with the corresponding internal control protein band supported for Fig. 2. **Figure S4.** 3D185 reversed M2-like macrophage-induced CD8^+^ T cell suppression. **Figure S5.** Analysis of body weight for tumor-bearing mice and Ki67, CD31expression as well as tumor infiltration CD8^+^ T and MDSC cell in tumor models. (DOCX 8.29 mb)

## Data Availability

All data and materials from the current study will be provided by the corresponding author on reasonable request.

## Abbreviations

CCK-8: Cell Counting Kit-8
CFDA: China Food and Drug Administration
CSF-1R: Colony-stimulating factor 1 receptor
FGFR: Fibroblast growth factor receptor
HUVEC: Human umbilical vein endothelial cell
KDR: Vascular endothelial growth factor receptor 2
MDSCs: Myeloid-derived suppressor cells
PDGFR: Platelet-derived growth factor receptor
RTK: Receptor tyrosine kinase
SRB: Sulforhodamine B
TAMs: Tumor-associated macrophages
