# Supplementary material for: Preclinical evaluation of 3D185, a novel potent inhibitor of FGFR1/2/3 and CSF-1R, in FGFR-dependent and macrophage-dominant cancer models
Source: J Exp Clin Cancer Res. 2019 Aug 22;38:372. doi: 10.1186/s13046-019-1357-y (PMC6704710; doi:10.1186/s13046-019-1357-y)
Supplement: Supplementary file 1 — Table S1. Some background information of cell lines used in the study. Table S2. Enzymatic kinase activity of the 3D185 and AZD4547. Table S3. Profiling of 3D185 against 372 kinases by Eurofins. Table S4. Anti-proliferative activity of 3D185 in sensitive cell lines. Table S5. Antiproliferative assay used in indicated cell lines in Fig. 2. Table S6. The effect of 3D185 and PLX3397 on cell survival of CSF-1-differentiated or GM-CSF-differentiated murine and human ‘protumor’ macrophages or ‘antitumor’ Mo-DCs/macrophages. Figure S1. The gating strategies for Macrophage, CD8+ T cell, Treg in flow cytometry analyses of tumor-infiltrating immune cells. Figure S2. 3D185 inhibited FGFR1 kinase activity in an ATP-competitive manner and inhibited bFGF-stimulated FGFR signaling in primary HUVECs. Figure S3. The intensity of phosphorylated protein band was quantified and normalized with the corresponding internal control protein band supported for Fig. 2. Figure S4. 3D185 reversed M2-like macrophage-induced CD8+ T cell suppression. Figure S5. Analysis of body weight for tumor-bearing mice and Ki67, CD31expression as well as tumor infiltration CD8+ T and MDSC cell in tumor models. (DOCX 8.29 mb) [file 13046_2019_1357_MOESM1_ESM.docx]

**Preclinical evaluation of 3D185, a novel potent inhibitor of FGFR1/2/3 and CSF1R, in FGFR-dependent and macrophage-dominant cancer models**

Xia Peng, Pengcong Hou, Yi Chen, Yang Dai, Yinchun Ji, Yanyan Shen, Yi Su, Bo Liu, Yueliang Wang, Deqiao Sun, Yuchen Jiang, Chuantao Zha, Zuoquan Xie, Jian Ding, Meiyu Geng, Jing Ai

**Supplementary materials and methods**

**Western blot analysis**

Cells were treated with the indicated doses of the compounds for 2 h at 37 °C and then lysed in 1× SDS sample buffer. If growth factor treatment was required, the cells were starved in serum-free medium for 24 h and then treated with the compounds and recombinant human/mouse growth factors for the appropriate period of time. Cell lysates were subsequently analyzed by Western blotting, as described previously [[1](#_ENREF_1)].

The intensity of phosphorylated protein band was quantified and normalized with the corresponding internal control protein band using ImageJ Software.

**Cell proliferation and viability assays**

The cancer cell lines indicated in Figure 3 were seeded in 96-well plates at a low density in growth medium. The next day, appropriate controls or the designated concentrations of the compounds were added to each well, and the cells were incubated for 72 h. HUVECs (passage 3) were seeded in 96-well plates in growth medium overnight and transferred to serum-free medium for 24 h. The following day, appropriate controls or the designated concentrations of the compounds were added to each well, and 100 ng/mL growth factor (bFGF or VEGF) was added to the indicated wells. The cells were incubated for 48 h. Finally, cell proliferation was determined using a sulforhodamine B (SRB) assay or a cell counting kit-8 (CCK-8) assay. The proliferation inhibition rate (IR) was calculated with the following formula: IR (%) = (OD_control_-OD_treated_) / OD_control_×100%. IC_50_ values for proliferation curves were calculated using a 4-parameter logistic nonlinear regression model with SoftMax Pro Software (Molecular Devices, San Jose, USA).

Murine bone marrow-derived macrophages (BMDMs) and PBMCs were used for cell viability studies. BMDMs were harvested by flushing the tibias and femurs of 6- to 8-week-old C57BL/6 mice and depleting red blood cells (RBCs) by RBC lysis (Biogems). For BMDM survival, the cells were cultured for 7 days in RPMI 1640 containing 10% fetal bovine serum (FBS), 100 U/mL penicillin, 100 μg/mL streptomycin and 100 ng/mL mouse CSF-1 (or CSF-2) with or without the tested compounds. PBMCs were obtained from HemaCare and cultured for 7 days in RPMI 1640 containing 10% fetal bovine serum (FBS), 100 U/mL penicillin, 100 μg/mL streptomycin and 100 ng/mL human CSF-1 (or CSF-2) with or without the tested compounds. Cell viability was measured using the CCK-8 assay. For IL-4/IL-13-induced macrophage survival analysis, viable cells were counted by trypan blue exclusion.

**Immunohistochemistry (IHC)**

Tumor specimens were fixed in 4% paraformaldehyde for 24 h, embedded in paraffin, and sectioned for microscope slides. After dewaxing and blocking endogenous peroxidase activity with 3% H_2_O_2_, the sections were incubated with 1.5% normal goat serum and then with an antibody overnight at 4 °C. Next, the sections were incubated with biotin-conjugated anti-rabbit IgG for 2 h at 37 °C, followed by incubation with avidin-biotin-peroxidase complex (ABC) for 1 h using the Vectastain ABC Kit (Vector Laboratories). Staining was detected using the DAB (3,3’-diaminobenzidine tetrahydrochloride) Liquid System (ZSGB-Bio), and 3 different fields in each section were imaged. For IHC quantification, the number of positive brownstained cells over the total number of cells was estimated and used to determine the percentage (%) of staining area for each tumor marker with ImageJ Software.

**Pharmacodynamic studies**

To assess the pharmacodynamics of 3D185 in tumors, mice bearing established xenograft tumors were treated with a single dose of the compound (12.5, 25 or 50 mg/kg), and the tumors were harvested 6 h after drug administration. The tumors were snap-frozen in liquid nitrogen and then homogenized in 500 μL of protein extraction solution (radioimmunoprecipitation assay (RIPA) buffer). The tumor extracts were then subjected to Western blot analysis. Individual bands corresponding to phospho-ERK and ERK were imaged.

**Supplementary Table**

Table S1. Some background information of cell lines used in the study

| Molecular Classification | Cell lines | Target gene aberration | Other gene  aberration | Cancer Type |
| --- | --- | --- | --- | --- |
| FGFR | KG1 | FGFR10P2-FGFR1 translocation | TP53 Splice Site | acute myelogenous leukemia |
|  | H1581 | FGFR1 amplification | / | lung cancer |
|  | NCI-H520 |  | / | squamous cell lung carcinoma |
|  | NCI-H2444 |  | KRAS G12V  TP53 Y236C  PTEN P96S | NSCLC |
|  | SNU16 | FGFR2 amplification | MYC amplification  TP53 Y205F | stomache cancer |
|  | NCI-H716 |  | TP53 E224D  MYC amplification | colorectal cancer |
|  | KATOIII |  | TP53 DEL  GATA3 A395T  DDR2 R341Q | stomache cancer |
|  | SUM52PE |  | CDKN2A A68V | triple negative breast cancer |
|  | RT112 | FGFR3 amplification | TP53 R175H R248Q  MYC L82F  TERT(promotor) | bladder cacner |
|  | UMUC14 | FGFR3 S249C mutation | TERT(promotor) | bladder cacner |
|  | OPM2 | t(4,14)FGFR3 and FGFR3 mutation | TP53 R175H  CDNK2A H83Y  TERT SLIENT | multiple myeloma |
|  | HUH7 | FGF19 amplification | TP53 Y220C  ROS1 S1109A | hepatocellular carcinoma |
| EGFR | NCI-H1975 | L858R, T790M | TP53 R273H  PIK3CA G118D  PDGFRA SILENT | lung adenocarcinoma |
| MET | EBC-1 | Amplification | DDR2 T681I | squamous cell lung cancer |
|  | MKN45 |  | TP53 wt/R110C  EGFR A1048V | gastric cancer |
| ROS1 | HCC78 | *SLC34A2-ROS1* | TP53 S241F  ALK R753Q  MYC E236K | NSCLC |
| KARS | NCI-H460 | Q61H | PIK3CA E545K | large cell lung cancer |
| NRAS | NCI-H1299 | Q61K | / | NSCLC |
| BRAF | A375 | V600E | MTOR R2152C  CDKN2A E69* E61* | Amelanotic melanoma |
| HER2/c-erb-2 | SK-BR-3 | amplification | CDH1 DEL  TP53 A175H | Breast adenocarcinoma |
| Others | HCC366 |  | P53 Y220C  HRAS slient G115G  DDR2 L239R  PIK3R1 D13H | Adenosquamous lung carcinoma |
|  | MKN1 |  | PIK3CA E545K  TP53 V143A  MYCPB2 V1674A | Gastric adenosquamous carcinoma |
|  | MKN28 |  | TP53 I251L  PIK3CA E545K  PIK3R4 L480L  MYCBP2 V1674A | Gastric tubular adenocarcinoma |
|  | SGC-7901 |  | / | Gastric cancer |
|  | BGC-823 |  | / | Gastric cancer |
|  | HCC1954 |  | PIK3CA H1047R  TP53Y163C  RRAS2 V202V | Ductal breast carcinoma |
|  | MCF-7 |  | CDKN2A DEL  PIK3CA E545K  WNT7B | breast adenocarcinoma |
|  | T47D |  | PIK3CA H1047R  TP53 L194F | Ductal breast carcinoma |
|  | MDA-MB-453 |  | AR Q868H  CDH1  KRAS G13D  PIK3CA H1047R  PTEN E307K | Breast adenocarcinoma |
|  | CAL51 |  | ALK K1109N  PIK3CA E542K  PTEN TK321fs  PTEN DEL E288fs | Breast carcinoma |
| CSF-1R | M-NFS-60 | M-CSF Dependent | _/_ | Mouse myelogenous leukemia |

The above information in the table was obtained based on the public Cancer Cell Line Encyclopedia (CCLE) database, Catalogue of Somatic Mutations in Cancer (COSMIC) database, SIB Bioinformatics Resource Portal (ExPASy) database, and references [[2-15](#_ENREF_2)] .

Table S2. Enzymatic kinase activity of the 3D185 and AZD4547

| Kinase | IC_50_ (nM) | |
| --- | --- | --- |
|  | 3D185 | AZD4547 |
| FGFR1 | 0.5±0.1 | 1.3±0.2 |
| FGFR2 | 1.3±0.1 | 1.8±0.3 |
| FGFR3 | 3.6±0.4 | 4.8±1.1 |
| FGFR4 | 51.4±6.9 | 150.3±21.0 |
| CSF-1R* | 3.8±0.4 | 40.3±1.3 |
| VEGFR2 | 381.5±8.9 | 83.7±2.6 |
| VEGFR1 | >1000 | / |
| VEGFR3 | >1000 | / |
| PDGFRα | >1000 | / |
| PDGFRβ | >1000 | / |

IC_50_ values are presented as the mean ± SD.

*the activity of PLX3397 against CSF1R IC_50_=1.4 ± 0.3 nM

Table S3. Profiling of 3D185 against 372 kinases by Eurofins

| Concentration (nM)  Kinases | 10 | 100 | 1000 |
| --- | --- | --- | --- |
|  | Inhibition rate (%) | | |
| FGFR1(h) | 99 | 98 | 100 |
| FGFR1(V561M)(h) | 58 | 94 | 100 |
| FGFR2(h) | 93 | 98 | 100 |
| FGFR2(N549H)(h) | 96 | 98 | 100 |
| FGFR3(h) | 94 | 99 | 100 |
| FGFR4(h) | 43 | 91 | 100 |
| Fms(h) | 75 | 93 | 96 |
| Fms(Y969C)(h) | 34 | 75 | 95 |
| Abl(h) | 3 | 43 | 81 |
| ACK1(h) | 1 | 37 | 90 |
| ACTR2(h) | 0 | -1 | 1 |
| ALK(h) | -1 | 35 | 82 |
| ALK1(h) | 3 | -7 | -1 |
| ALK2(h) | -10 | -15 | -4 |
| ALK4(h) | -4 | -7 | 5 |
| ALK6(h) | 3 | -11 | -5 |
| Arg(h) | 2 | 36 | 87 |
| AMPKα1(h) | -8 | -6 | -13 |
| AMPKα2(h) | -4 | -8 | 9 |
| A-Raf(h) | -7 | -7 | -4 |
| ARK5(h) | 11 | 16 | 13 |
| ASK1(h) | -6 | -7 | -2 |
| Aurora-A(h) | 4 | 5 | 3 |
| Aurora-B(h) | 7 | -4 | 5 |
| Aurora-C(h) | -15 | 6 | -2 |
| Axl(h) | -7 | -9 | 26 |
| Blk(h) | -18 | 16 | 88 |
| BMPR2(h) | 5 | -2 | 4 |
| BRK(h) | -5 | 7 | 46 |
| BrSK1(h) | 13 | 11 | 11 |
| BrSK2(h) | 8 | 5 | 11 |
| BTK(h) | 11 | 39 | 90 |
| BTK(R28H)(h) | -3 | -2 | -4 |
| B-Raf(h) | -4 | -5 | 5 |
| B-Raf(V599E)(h) | -1 | -6 | 7 |
| CaMKI(h) | 11 | 14 | 64 |
| CaMKIß(h) | -2 | 6 | 32 |
| CaMKIγ(h) | -1 | 3 | 41 |
| CaMKIIα(h) | -9 | 2 | -15 |
| CaMKIIβ(h) | -1 | -3 | -4 |
| CaMKIIγ(h) | -2 | -6 | -9 |
| CaMKIδ(h) | 0 | 23 | 76 |
| CaMKIIδ(h) | -1 | -3 | -3 |
| CaMKIV(h) | -6 | -21 | -22 |
| CaMKK1(h) | -1 | 5 | 35 |
| CaMKK2(h) | -4 | -22 | 4 |
| Cdc7/cyclinB1(h) | 5 | -5 | 8 |
| CDK1/cyclinB(h) | 0 | 2 | -11 |
| CDK2/cyclinA(h) | 2 | 7 | -3 |
| CDK2/cyclinE(h) | -5 | 2 | 10 |
| CDK3/cyclinE(h) | 1 | -11 | -19 |
| CDK4/cyclinD3(h) | 5 | -4 | 11 |
| CDK5/p25(h) | 10 | 4 | 3 |
| CDK5/p35(h) | 19 | 17 | 12 |
| CDK6/cyclinD3(h) | 9 | 15 | 16 |
| CDK7/cyclinH/MAT1(h) | 12 | 11 | 11 |
| CDK9/cyclin T1(h) | -12 | 0 | 4 |
| CDK12/cyclinK(h) | -6 | 4 | 7 |
| CDK13/cyclinK(h) | -16 | 8 | 1 |
| CDK18/cyclinY(h) | 10 | 12 | 15 |
| CDKL1(h) | 3 | -5 | 0 |
| CDKL3(h) | -12 | -17 | -9 |
| ChaK1(h) | 2 | -10 | 8 |
| CHK1(h) | 3 | -6 | -3 |
| CHK2(h) | 0 | 4 | 11 |
| CHK2(I157T)(h) | -6 | -5 | -1 |
| CHK2(R145W)(h) | -17 | -15 | -4 |
| CK1γ1(h) | 9 | -4 | 2 |
| CK1γ2(h) | -9 | -8 | -7 |
| CK1γ3(h) | 1 | -10 | 12 |
| CK1δ(h) | -10 | 0 | 6 |
| CK1(y) | -1 | 0 | 2 |
| CK2(h) | 2 | 9 | 9 |
| CK2α1(h) | 23 | 21 | 10 |
| CK2α2(h) | 0 | 1 | -6 |
| CLIK1(h) | 0 | -1 | 12 |
| CLK1(h) | -7 | -5 | 7 |
| CLK2(h) | 5 | 3 | 7 |
| CLK3(h) | 2 | 9 | 16 |
| CLK4(h) | 5 | 19 | 27 |
| cKit(h) | 19 | 35 | 76 |
| cKit(D816V)(h) | -7 | 1 | 3 |
| cKit(D816H)(h) | 22 | 27 | 44 |
| cKit(V560G)(h) | 10 | 38 | 71 |
| cKit(V654A)(h) | 4 | 14 | 37 |
| CSK(h) | 8 | 23 | 63 |
| c-RAF(h) | 13 | 14 | 5 |
| cSRC(h) | 26 | 53 | 87 |
| DAPK1(h) | 5 | 13 | 19 |
| DAPK2(h) | 1 | -8 | 2 |
| DCAMKL2(h) | 8 | 18 | 28 |
| DCAMKL3(h) | 5 | 7 | 16 |
| DDR1(h) | 6 | 27 | 64 |
| DDR2(h) | 0 | 7 | 54 |
| DMPK(h) | 2 | 3 | 2 |
| DRAK1(h) | -4 | 7 | 14 |
| DRAK2(h) | -5 | -13 | 3 |
| DYRK1A(h) | -4 | 1 | 5 |
| DYRK1B(h) | 12 | 3 | 3 |
| DYRK2(h) | -1 | -1 | 12 |
| DYRK3(h) | -24 | -31 | -27 |
| eEF-2K(h) | 4 | 2 | 11 |
| EGFR(h) | -12 | -16 | 0 |
| EGFR(L858R)(h) | 1 | 9 | 30 |
| EGFR(L861Q)(h) | -5 | -5 | 39 |
| EGFR(T790M)(h) | -15 | -8 | 29 |
| EGFR(T790M,L858R)(h) | 6 | 38 | 79 |
| EphA1(h) | 0 | -14 | -6 |
| EphA2(h) | 13 | 24 | 73 |
| EphA3(h) | 2 | 14 | 30 |
| EphA4(h) | 14 | 5 | 13 |
| EphA5(h) | 11 | 10 | 13 |
| EphA7(h) | 0 | 8 | 21 |
| EphA8(h) | 1 | 5 | 29 |
| EphB2(h) | 12 | 6 | 15 |
| EphB1(h) | -9 | 1 | 36 |
| EphB3(h) | 13 | 1 | 5 |
| EphB4(h) | 8 | -5 | 2 |
| ErbB2(h) | -7 | -11 | -11 |
| ErbB4(h) | -2 | -1 | 28 |
| FAK(h) | 17 | 29 | 58 |
| Fer(h) | -11 | 0 | 42 |
| Fes(h) | -8 | 36 | 91 |
| Fgr(h) | 11 | 44 | 89 |
| Flt3(D835Y)(h) | -1 | -6 | 5 |
| Flt3(h) | 10 | 17 | 27 |
| Fyn(h) | 8 | 27 | 80 |
| GCN2(h) | 3 | -12 | -49 |
| GRK1(h) | -2 | -5 | -7 |
| GRK2(h) | 5 | 1 | -4 |
| GRK3(h) | 6 | 3 | 3 |
| GRK5(h) | -1 | -9 | 3 |
| GRK6(h) | -4 | -6 | -5 |
| GRK7(h) | 9 | 16 | 27 |
| GSK3α(h) | 10 | 4 | -5 |
| GSK3β(h) | 0 | 5 | -5 |
| Haspin(h) | -15 | -6 | 4 |
| Hck(h) activated | 6 | 44 | 87 |
| HIPK1(h) | -3 | 4 | 10 |
| HIPK2(h) | 3 | 6 | -10 |
| HIPK3(h) | -2 | -12 | -8 |
| HIPK4(h) | 1 | -17 | 16 |
| HPK1(h) | 17 | 65 | 94 |
| ICK(h) | -10 | -5 | 4 |
| IGF-1R(h) | -5 | 54 | 88 |
| IGF-1R(h), activated | -12 | 6 | 49 |
| IKKα(h) | -3 | -3 | -7 |
| IKKβ(h) | 0 | -5 | 15 |
| IKKε(h) | -4 | 2 | 5 |
| IR(h) | 16 | 32 | 94 |
| IR(h), activated | 9 | 42 | 85 |
| IRE1(h) | -1 | -13 | 6 |
| IRR(h) | -5 | 5 | 1 |
| IRAK1(h) | 4 | 0 | 19 |
| IRAK4(h) | -2 | -2 | -4 |
| JAK1(h) | 13 | 15 | 20 |
| JAK2(h) | -14 | 5 | 6 |
| JAK3(h) | -11 | 0 | 10 |
| JNK1α1(h) | -3 | -4 | 3 |
| JNK2α2(h) | 3 | 4 | 1 |
| JNK3(h) | -17 | -22 | -36 |
| KDR(h) | -2 | 1 | 10 |
| Lck(h) | 22 | 60 | 94 |
| Lck(h) activated | 16 | 59 | 92 |
| LIMK1(h) | 2 | 8 | 0 |
| LKB1(h) | -9 | 7 | 2 |
| LOK(h) | 1 | 27 | 77 |
| Lyn(h) | 8 | 81 | 98 |
| LRRK2(h) | -10 | -3 | 21 |
| LTK(h) | -8 | 1 | 37 |
| MAK(h) | -1 | -9 | -17 |
| MAPK1(h) | -5 | -6 | 0 |
| MAPK2(h) | -5 | -3 | 3 |
| MAPKAP-K2(h) | 0 | 10 | 0 |
| MAPKAP-K3(h) | 8 | 17 | 19 |
| MEK1(h) | 10 | 14 | 8 |
| MEK2(h) | -18 | -19 | 8 |
| MARK1(h) | -11 | -13 | -25 |
| MARK3(h) | 7 | -6 | -4 |
| MARK4(h) | 3 | 5 | -2 |
| MEKK2(h) | 34 | 85 | 97 |
| MELK(h) | -1 | 8 | 14 |
| Mer(h) | -9 | -7 | 32 |
| Met(h) | 16 | 15 | 55 |
| Met(D1246N)(h) | -18 | -11 | 3 |
| Met(M1268T)(h) | -12 | -11 | 15 |
| Met(Y1248C)(h) | -9 | -2 | 5 |
| Met(Y1248D)(h) | -12 | -1 | 18 |
| Met(Y1248H)(h) | -16 | 8 | 46 |
| MINK(h) | 28 | 68 | 90 |
| MKK6(h) | 11 | 0 | 2 |
| MKK7β(h) | 9 | -2 | 12 |
| MLCK(h) | 5 | -1 | 11 |
| MLK1(h) | -9 | -1 | 26 |
| MLK2(h) | 4 | 2 | 24 |
| Mnk2(h) | -2 | -10 | -9 |
| MOK(h) | -7 | 6 | 1 |
| MRCKα(h) | 12 | 16 | 14 |
| MRCKβ(h) | 18 | 8 | 21 |
| MSK1(h) | 31 | 19 | 24 |
| MSK2(h) | 10 | 25 | 49 |
| MSSK1(h) | 3 | 11 | 18 |
| MST1(h) | 2 | 11 | 32 |
| MST2(h) | 16 | 41 | 65 |
| MST3(h) | -2 | 8 | 70 |
| MST4(h) | 6 | 27 | 70 |
| mTOR(h) | -2 | -2 | 4 |
| mTOR/FKBP12(h) | 1 | 0 | 2 |
| MuSK(h) | 2 | 7 | 47 |
| MYLK2(h) | 7 | 0 | 21 |
| MYO3B(h) | -4 | 0 | 7 |
| NDR2(h) | 2 | -8 | 1 |
| NEK1(h) | 5 | 3 | 11 |
| NEK2(h) | 15 | 67 | 94 |
| NEK4(h) | 16 | 15 | 74 |
| NEK3(h) | 10 | 13 | 5 |
| NEK6(h) | -7 | 4 | 2 |
| NEK7(h) | 2 | 1 | -1 |
| NEK9(h) | -12 | -6 | 14 |
| NIM1(h) | -9 | 0 | 0 |
| NEK11(h) | 5 | 15 | 37 |
| NLK(h) | 0 | -6 | 3 |
| NUAK2(h) | -1 | -1 | 0 |
| p70S6K(h) | -4 | -9 | -1 |
| PAK1(h) | 5 | 6 | 2 |
| PAK2(h) | 5 | -5 | -5 |
| PAK4(h) | -8 | 0 | -2 |
| PAK3(h) | -1 | -3 | 7 |
| PAK5(h) | -11 | -2 | 5 |
| PAK6(h) | -3 | 12 | -4 |
| PAR-1Bα(h) | -4 | -3 | 2 |
| PASK(h) | -15 | 1 | 10 |
| PEK(h) | -7 | 6 | 55 |
| PDGFRα(h) | 9 | 6 | 10 |
| PDGFRα(D842V)(h) | -2 | 5 | 9 |
| PDGFRα(V561D)(h) | 5 | 23 | 57 |
| PDGFRβ(h) | 1 | -1 | 17 |
| PDHK4(h) | -2 | -2 | 0 |
| PDK1(h) | -5 | -14 | -4 |
| PhKγ1(h) | -1 | -9 | -12 |
| PhKγ2(h) | 12 | -8 | -3 |
| Pim-1(h) | -5 | 13 | 9 |
| Pim-2(h) | 11 | 3 | 6 |
| Pim-3(h) | -5 | -11 | -1 |
| PKA(h) | 5 | -8 | 5 |
| PKAcβ(h) | -6 | -11 | -11 |
| PKBα(h) | -6 | -8 | -7 |
| PKBβ(h) | 15 | 29 | 30 |
| PKBγ(h) | 4 | 12 | 20 |
| PKCα(h) | -1 | 1 | 3 |
| PKCβI(h) | -7 | -3 | -1 |
| PKCβII(h) | -12 | -1 | -8 |
| PKCγ(h) | -4 | -8 | -1 |
| PKCδ(h) | 5 | 4 | -1 |
| PKCε(h) | -4 | -10 | -6 |
| PKCη(h) | -10 | -1 | -9 |
| PKCι(h) | -1 | 0 | -1 |
| PKCμ(h) | -2 | 2 | 11 |
| PKCθ(h) | -3 | -4 | 1 |
| PKCζ(h) | -11 | 7 | -22 |
| PKD2(h) | 7 | -4 | 3 |
| PKD3(h) | -3 | -2 | 11 |
| PKG1α(h) | -1 | -2 | 1 |
| PKG1β(h) | 12 | 1 | 4 |
| PKR(h) | -4 | -3 | 0 |
| Plk1(h) | -6 | 2 | -4 |
| Plk3(h) | 5 | 1 | 6 |
| Plk4(h) | -18 | -20 | -17 |
| PRAK(h) | -18 | -24 | -10 |
| PRKG2(h) | 1 | 3 | 3 |
| PRK1(h) | 2 | 1 | 2 |
| PRK2(h) | 12 | 4 | 3 |
| PrKX(h) | 8 | 11 | 5 |
| PRP4(h) | -8 | -9 | -1 |
| PTK5(h) | 19 | 64 | 91 |
| Pyk2(h) | 2 | 24 | 71 |
| Ret(h) | 8 | 27 | 80 |
| Ret (V804L)(h) | -13 | -15 | 20 |
| Ret(V804M)(h) | -3 | -2 | 56 |
| RIPK1(h) | 4 | 86 | 99 |
| RIPK2(h) | 2 | 6 | 3 |
| ROCK-I(h) | -2 | -12 | -3 |
| ROCK-II(h) | 0 | -1 | -1 |
| ROCK-II(r) | -3 | 0 | -1 |
| Ron(h) | -16 | -10 | 28 |
| Ros(h) | -4 | 6 | 17 |
| Rse(h) | 5 | -2 | 16 |
| Rsk1(h) | -1 | -4 | 24 |
| Rsk1(r) | -17 | 8 | 30 |
| Rsk2(h) | -19 | 0 | 24 |
| Rsk3(h) | -17 | -19 | -15 |
| Rsk4(h) | 0 | 6 | 19 |
| SAPK2a(h) | -7 | -2 | -4 |
| SAPK2a(T106M)(h) | 0 | 3 | -3 |
| SAPK2b(h) | 3 | 14 | 20 |
| SAPK3(h) | -4 | -12 | -7 |
| SAPK4(h) | -12 | -8 | -11 |
| SBK1(h) | -13 | -3 | -10 |
| SGK(h) | -4 | -13 | -7 |
| SGK2(h) | 4 | 1 | 26 |
| SGK3(h) | 3 | 2 | 14 |
| SIK(h) | 4 | -17 | -5 |
| SIK2(h) | 7 | 5 | 4 |
| SIK3(h) | -4 | -1 | -2 |
| SLK(h) | 10 | 34 | 81 |
| Snk(h) | 0 | -2 | 10 |
| SNRK(h) | -8 | -42 | -44 |
| Src(1-530)(h) | 4 | 30 | 82 |
| Src(T341M)(h) | 8 | 33 | 88 |
| SRPK1(h) | 13 | 9 | 12 |
| SRPK2(h) | 2 | 2 | -7 |
| STK25(h) | 7 | 22 | 57 |
| STK32A(h) | -5 | 0 | 2 |
| STK32B(h) | 1 | 1 | 0 |
| STK32C(h) | 11 | 8 | 7 |
| STK33(h) | 3 | 9 | 13 |
| Syk(h) | -3 | -5 | 20 |
| TAF1L(h) | -4 | -9 | -9 |
| TAK1(h) | -6 | 11 | 13 |
| TAO1(h) | -2 | 6 | 21 |
| TAO2(h) | 12 | 28 | 66 |
| TAO3(h) | -1 | 7 | 36 |
| TBK1(h) | -6 | -2 | -1 |
| Tec(h) activated | 3 | 9 | 23 |
| TGFBR1(h) | -15 | -19 | -17 |
| TGFBR2(h) | -10 | -5 | -9 |
| Tie2 (h) | 19 | 64 | 95 |
| Tie2(R849W)(h) | -6 | 39 | 86 |
| Tie2(Y897S)(h) | 14 | 39 | 88 |
| TLK1(h) | -1 | -4 | -8 |
| TLK2(h) | 0 | -1 | -1 |
| TRB2(h) | -3 | -7 | -4 |
| TrkB(h) | 31 | 87 | 97 |
| TrkC(h) | 36 | 85 | 98 |
| TSSK1(h) | 5 | 2 | 13 |
| TSSK2(h) | 6 | 5 | -4 |
| TSSK3(h) | 14 | 5 | 5 |
| TSSK4(h) | -6 | -10 | -9 |
| TTBK1(h) | 2 | -7 | -6 |
| TTBK2(h) | -4 | -11 | -9 |
| TTK(h) | 5 | -2 | 12 |
| Txk(h) | 18 | 27 | 49 |
| TYK2(h) | 1 | 14 | 67 |
| ULK1(h) | -2 | 4 | 5 |
| ULK2(h) | 4 | 14 | 22 |
| ULK3(h) | -8 | 2 | -1 |
| VRK2(h) | 4 | 11 | 17 |
| Wee1(h) | -5 | -16 | 6 |
| Wee1B(h) | 9 | -2 | 9 |
| WNK1(h) | 3 | 2 | 16 |
| WNK2(h) | 0 | -1 | 22 |
| WNK3(h) | -7 | 12 | 71 |
| Yes(h) | 10 | 66 | 97 |
| ZAK(h) | 1 | -2 | 8 |
| ZAP-70(h) | 12 | 0 | 8 |
| ZIPK(h) | -3 | 0 | 2 |
| ATM(h) | -3 | -5 | -5 |
| ATR/ATRIP(h) | -4 | 10 | 6 |
| DNA-PK(h) | 5 | 2 | 0 |
| PI3 Kinase (p110β/p85α)(h) | 0 | 1 | 4 |
| PI3 Kinase (p120γ)(h) | 0 | 1 | 3 |
| PI3 Kinase (p110𝛿/p85α)(h) | 1 | -4 | 5 |
| PI3 Kinase (p110α/p85α)(h) | -1 | 1 | 2 |
| PI3 Kinase (p110α(E542K)/p85α)(h) | -2 | 2 | 2 |
| PI3 Kinase (p110α(H1047R)/p85α)(h) | 1 | 3 | -5 |
| PI3 Kinase (p110α(E545K)/p85α)(h) | -2 | -2 | 2 |
| PI3 Kinase (p110α/p65α)(h) | 3 | 2 | 0 |
| PI3KC2α(h) | -5 | 1 | 1 |
| PI3KC2γ(h) | -4 | -4 | 1 |
| PIP4K2α(h) | 1 | -2 | 4 |
| PIP5K1α(h) | -1 | -4 | -9 |
| PIP5K1γ(h) | 6 | 6 | 10 |

Table S4. Anti-proliferative activity of 3D185 in sensitive cell lines

| Cell Line | IC_50_ (nM) | |
| --- | --- | --- |
|  | 3D185 | AZD4547 |
| KG-1 | 2.0±0.4 | 3.0±0.3 |
| NCI-H1581 | 17.3±5.7 | 43.8±8.6 |
| SNU16 | 0.9±0.1 | 2.7±0.4 |
| NCI-H716 | 3.0±0.4 | 7.0±0.9 |
| KATOIII | 11.3±0.2 | 15.1±2.9 |
| SUM52PE | 2.0±0.0 | 15.5±2.4 |
| RT112 | 36.8±6.8 | 83.3±6.4 |
| UMUC14 | 30.9±5.3 | 52.9±4.6 |
| OPM2 | 19.2±4.3 | 33.0±7.6 |
| HUH7 | 49.8±1.5 | 284.4±22.5 |
| M-NFS-60 | 16.7±2.5 | 1233.8±184.5 |

IC_50_ values are presented as the mean ± SD.

Table S5. Antiproliferative assay used in indicated cell lines in Figure 2

| Cell Line | Method | Cell Line | Method |
| --- | --- | --- | --- |
| KG1 | CCK8 | EBC-1 | SRB |
| H1581 | CCK8 | HCC336 | SRB |
| SNU16 | CCK8 | HCC78 | SRB |
| NIC-H716 | CCK8 | MKN45 | SRB |
| KATOIII | CCK8 | MKN1 | SRB |
| SUM52PE | SRB | MKN28 | SRB |
| RT112 | CCK8 | SGC-7901 | SRB |
| UMUC14 | SRB | BGC-823 | SRB |
| OPM2 | CCK8 | HCC1954 | SRB |
| HUH7 | SRB | MCF-7 | SRB |
| M-NFS-60 | CCK8 | T47D | SRB |
| NCI-H2444 | SRB | SK-BR-3 | SRB |
| NCI-H520 | SRB | MDB-MB-453 | SRB |
| NCI-H1975 | SRB | CAL51 | SRB |
| NCI-H460 | SRB | A375 | SRB |
| NCI-H1299 | SRB |  |  |

Table S6. The effect of 3D185 and PLX3397 on cell survival of CSF-1-differentiated or GM-CSF-differentiated murine and human ‘protumor’ macrophages or ‘antitumor’ Mo-DCs/macrophages

| Cell line | IC_50_ (nM) | |  |
| --- | --- | --- | --- |
|  | 3D185 | PLX3397 | AZD4547 |
| BMDM (CSF-1) | 57.8±13.5 | 194.9±48.2 | 883.0±132.6 |
| BMDM (CSF-2) | >1000 | >1000 | >1000 |
| PBMC (CSF-1) | 118.5±12.0 | 220.2±38.6 | / |
| PBMC (CSF-2) | >1000 | >1000 | / |

IC_50_ values are presented as the mean ± SD.


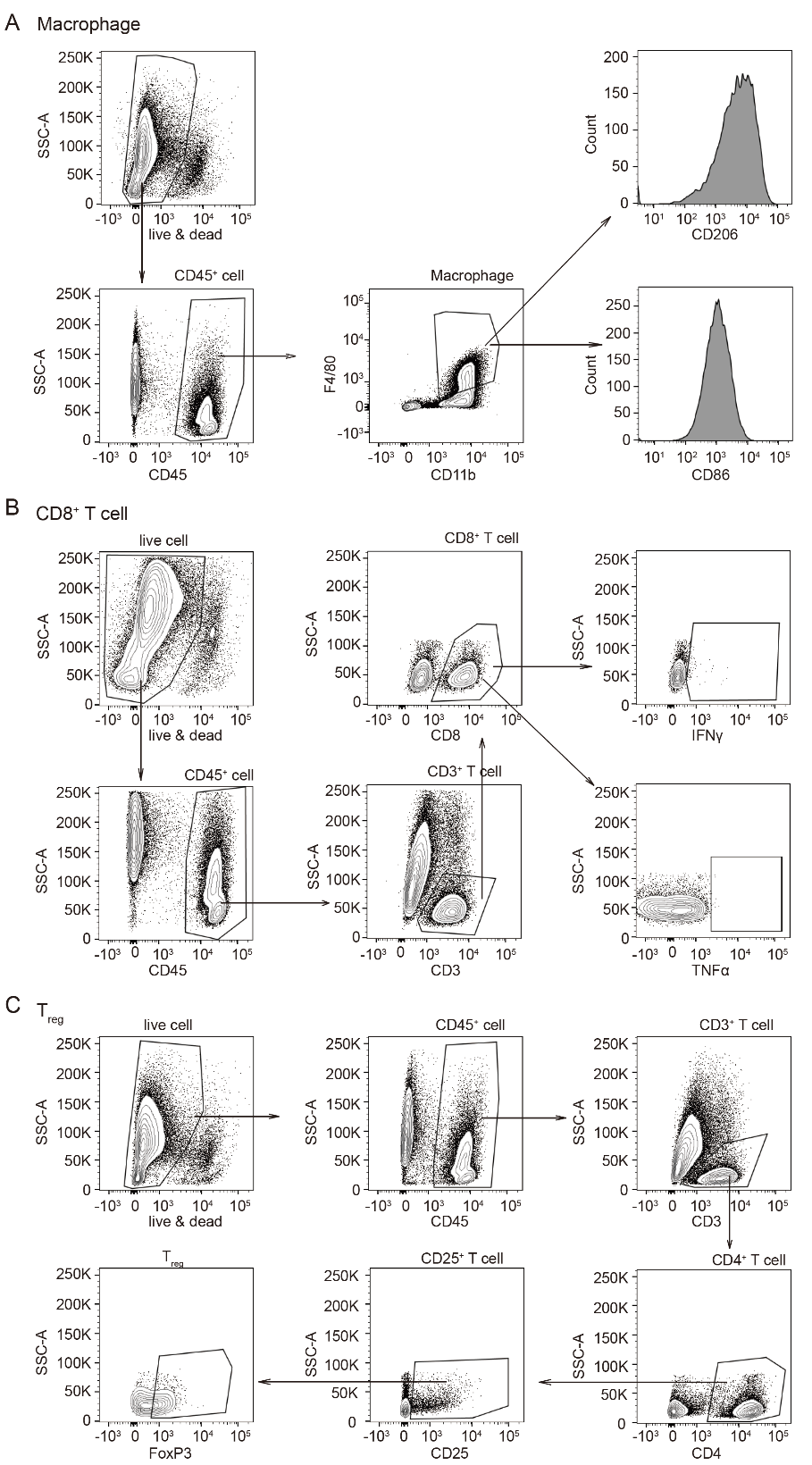
**Supplementary Figures and Figure Legends**

Figure S1. The gating strategies for Macrophage (A), CD8^+^T cell (B), T_reg_ (C) in flow cytometry analyses of tumor-infiltrating immune cells.


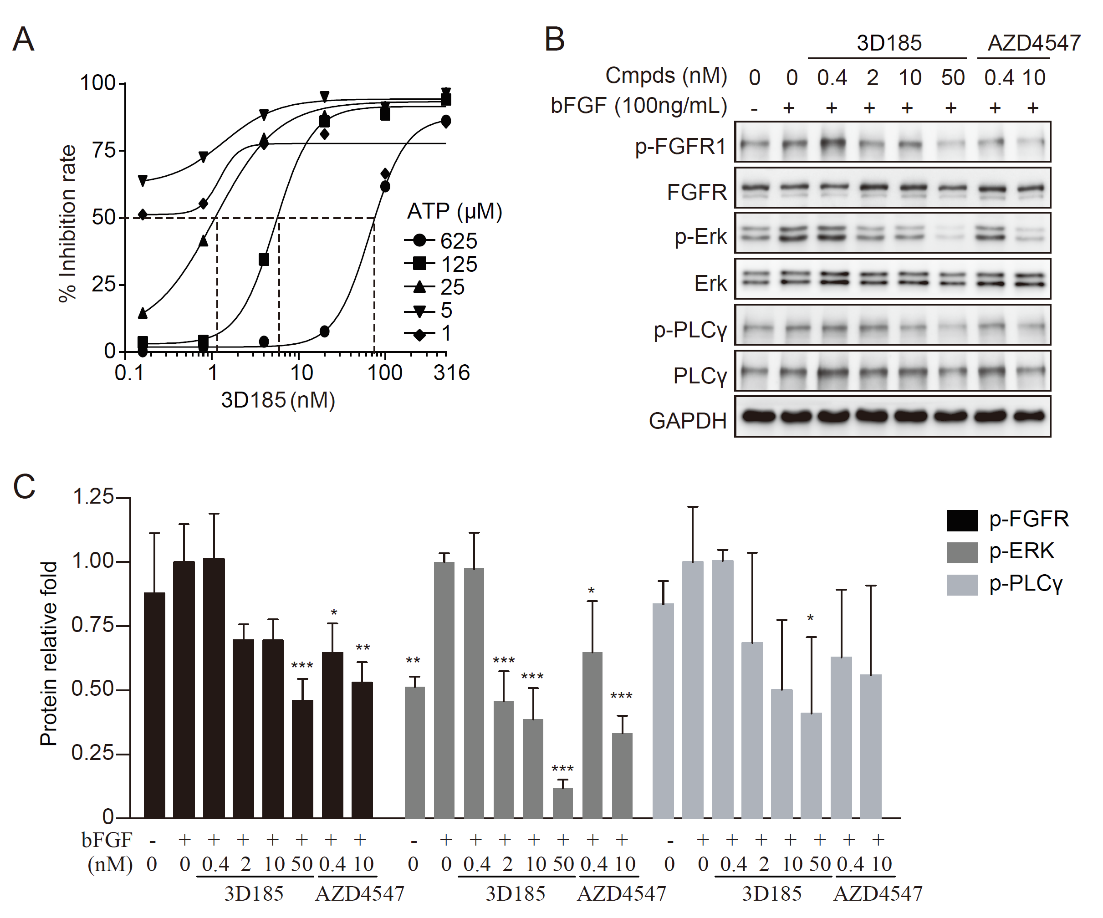


Figure S2. 3D185 inhibited FGFR1 kinase activity in an ATP-competitive manner and inhibited bFGF-stimulated FGFR signaling in primary HUVECs. A, ATP-competitive inhibition of FGFR1 kinase activity by 3D185. B-C, 3D185 inhibited bFGF-stimulated FGFR signaling in primary HUVECs. 3D185 suppresses bFGF-stimulated FGFR signaling (B), the intensity of phosphorylated protein band was quantified and normalized with the corresponding GAPDH band. Values are mean ± SD from triplicate experiments (C). Pre-starved primary HUVECs were treated with 3D185 for 2 h, stimulated with 100 ng/mL bFGF for 15 min, lysed and subjected to Western blot analysis.


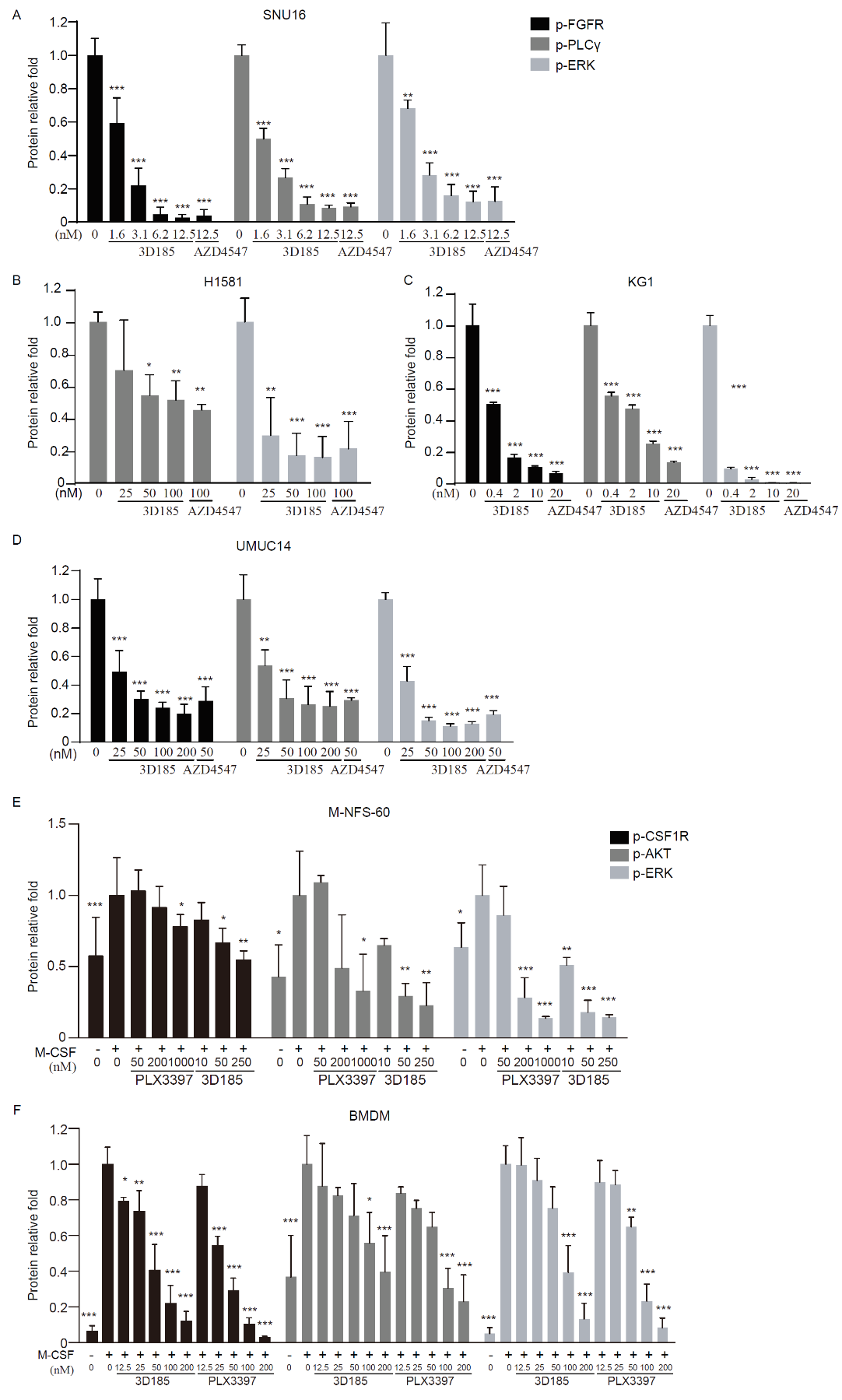


Figure S3. The intensity of phosphorylated protein band was quantified and normalized with the corresponding internal control protein band supported for Figure 2. Values are mean ± SD from triplicate experiments.


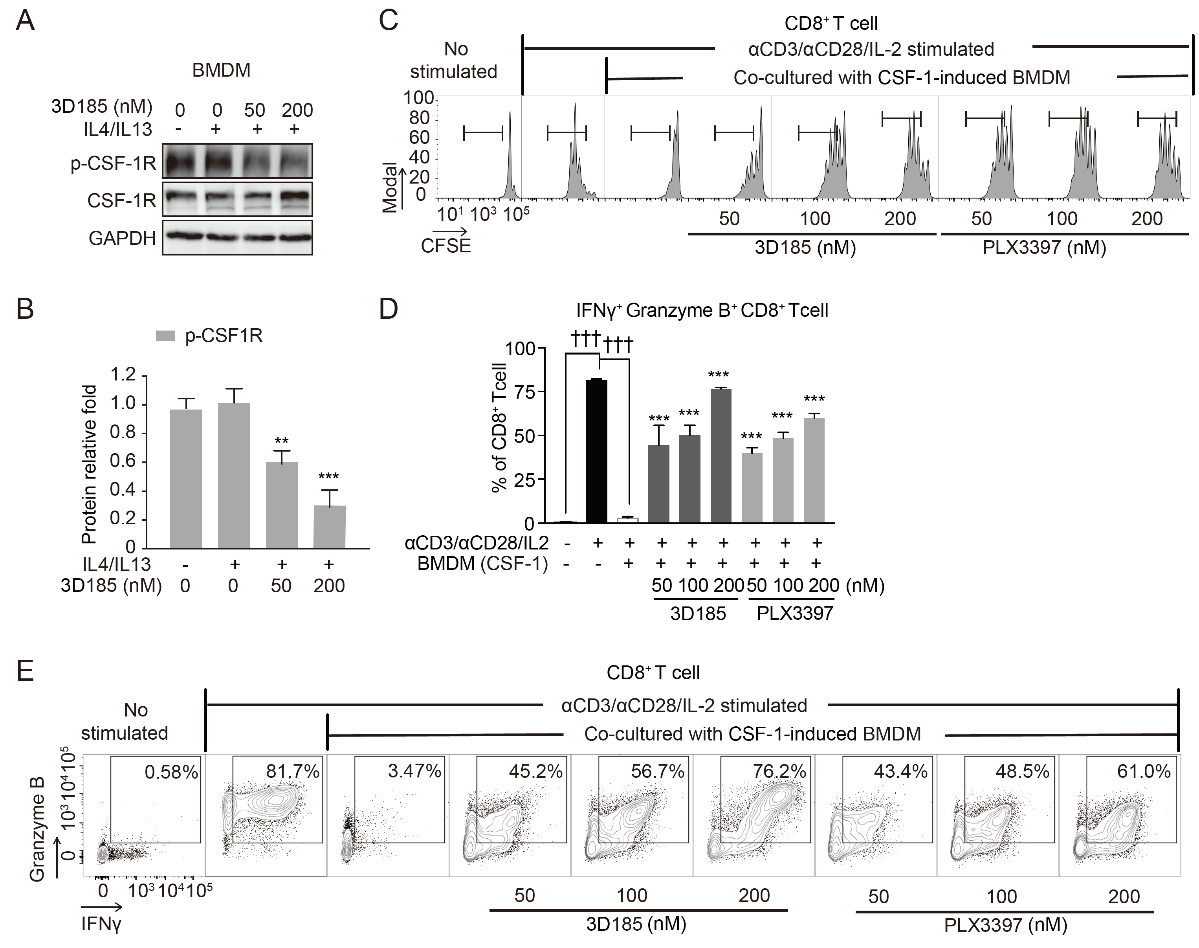


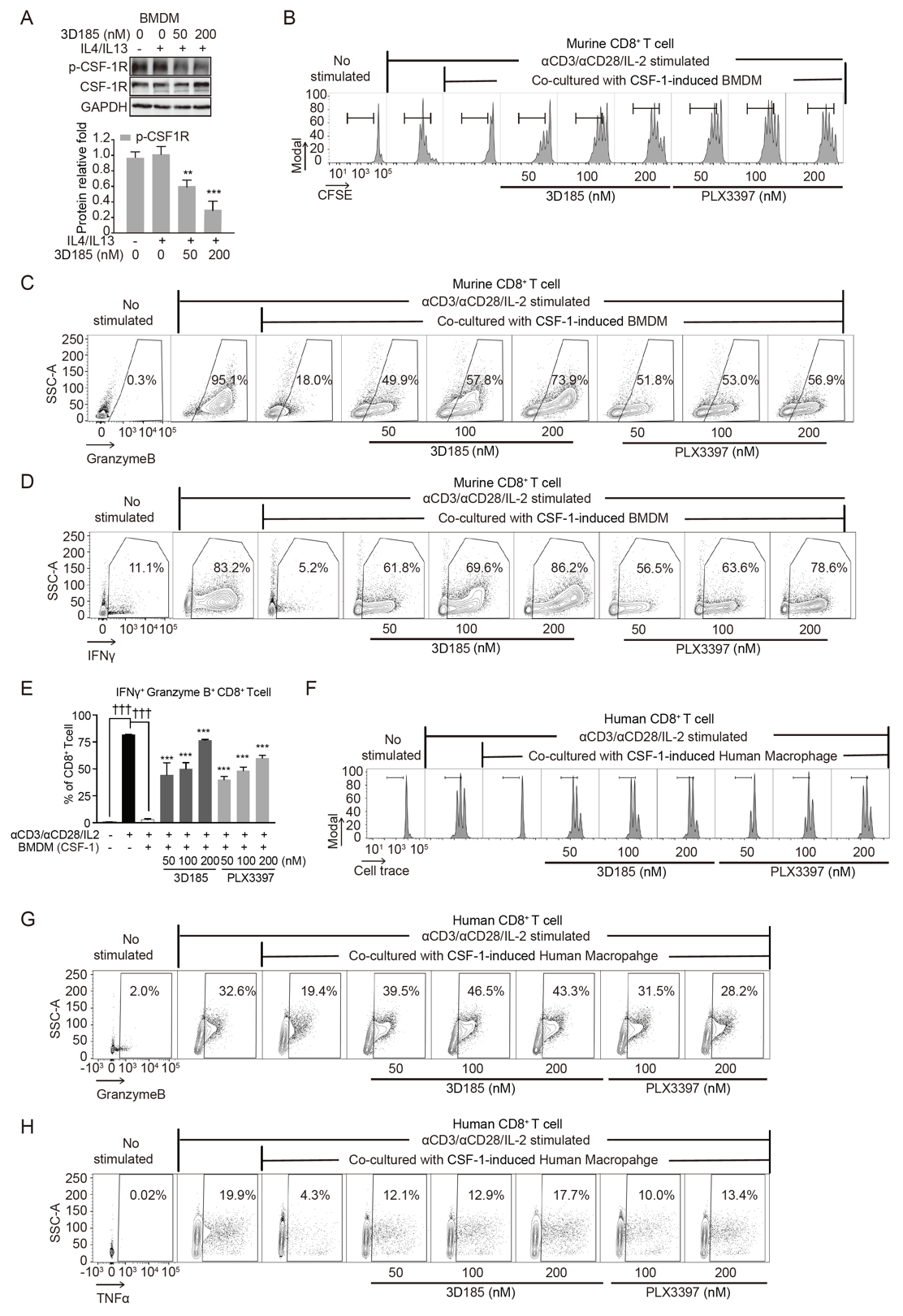


Figure S4. 3D185 reversed M2-like macrophage-induced CD8+ T cell suppression. A, 3D185 suppresses CSF-1R phosphorylation, the intensity of phosphorylated protein band was quantified and normalized with the corresponding GAPDH band. Values are mean ± S.D. from triplicate experiment. B-E, 3D185 reversed M2-like mac rophage-induced CD8^+^ T cell suppression. Representative CFSE images, granzyme B^+^ CD8^+^ T cells and IFN-γ^+^ CD8^+^ T cells image are presented in B, C and D. Quantification of IFN-γ^+^ granzyme B^+^ double positive CD8^+^ T cells in total CD8^+^ T cells (E). Data are shown as the mean ± SD. Significant differences were determined using one-way ANOVA with Tukey’s multiple-comparison test (*p < 0.05; **p < 0.01; ***p < 0.001). Murine bone marrow cells were induced to M2-like macrophages with CSF-1 for 7 days and then treated with 3D185 or PLX3397 for 48 h. Then, murine BMDMs were cocultured with CFSE-labeled spleen cells and stimulated with anti-CD3/CD28 beads and IL-2 for 72 h. Representative data from two independent experiments are shown. F-H, 3D185 reversed Human M2-like macrophage-induced CD8^+^ T cell suppression.Representative CFSE images, granzyme B^+^ CD8^+^ T cells and TNF-α^+^ CD8^+^ T cells image are presented in F, G and H. Data are shown as the mean ± SD. Significant differences were determined using one-way ANOVA with Tukey’s multiple-comparison test (*p < 0.05; **p < 0.01; ***p < 0.001). Human PBMC cells were induced to M2-like macrophages with CSF-1 for 7 days and then treated with 3D185 or PLX3397 for 48 h. Then, Human macrophages were cocultured with CFSE-labeled Human CD8^+^ T cells and stimulated with anti-CD3/CD28 beads and IL-2 for 72 h. Representative data from two independent experiments are shown.


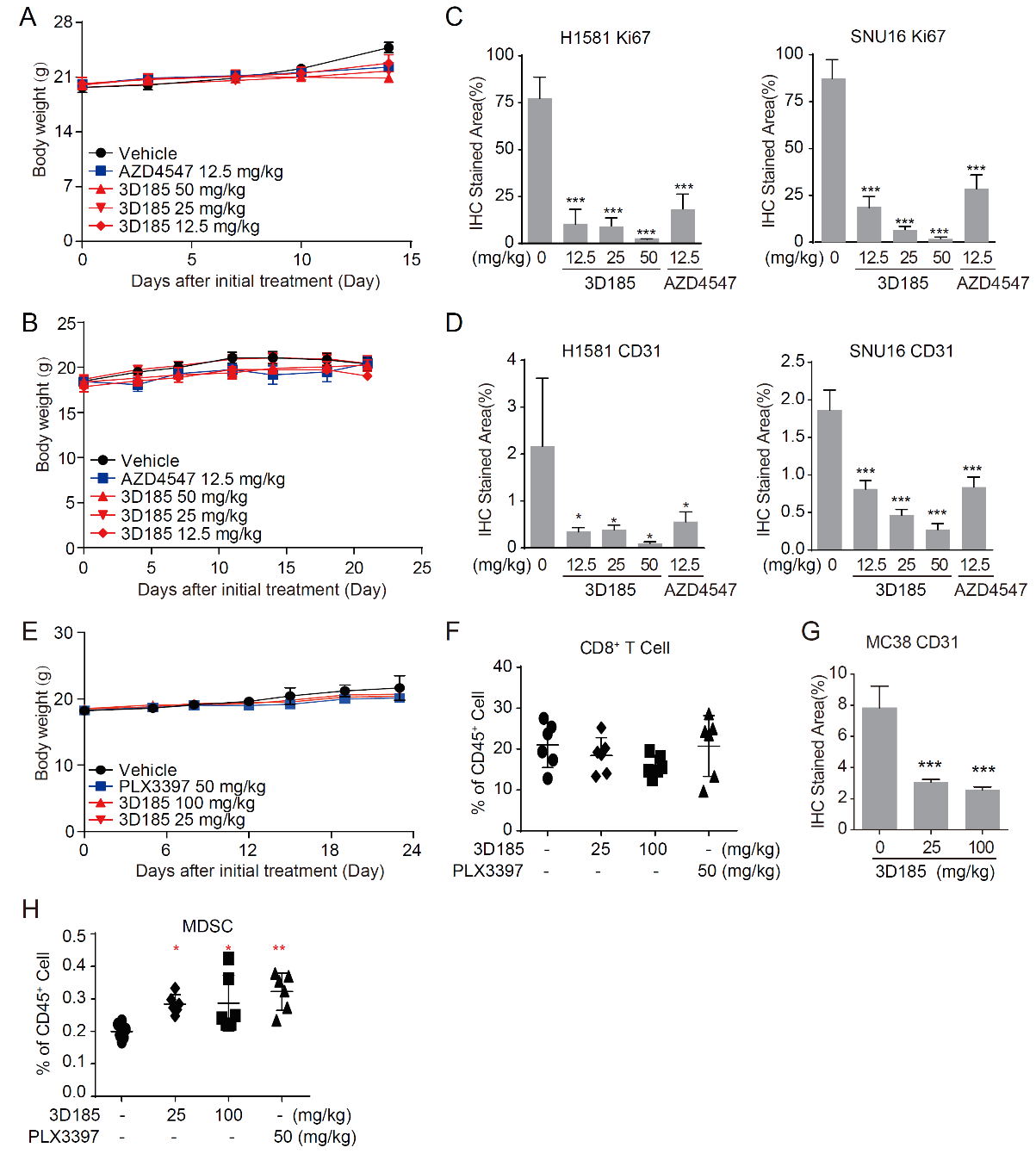


Figure S5. Analysis of body weight for tumor-bearing mice and Ki67, CD31expression as well as tumor infiltration CD8+T and MDSC cell in tumor models. A-B, Animal weights for 3D185-treated NCI-H1581 (A) and SNU16 (B) xenografts models. The weights are presented as the mean ± SEM during 3D185 treatment. C-D, Percentage of the stained areas of Ki67 (C) and CD31 (D) in NCI-H1581 and SNU16 tumor. Positive areas per field are expressed as means ± SD. *p < 0.05; **p < 0.01; ***p < 0.001, compared with vehicle group. E, Animal weights for 3D185-treated MC38 xenografts models. The weights are presented as the mean ± SEM during 3D185 treatment. F, Flow cytometric analysis of the infiltration of CD8+ T cells in the MC38 tumor model treated with vehicle or the indicated inhibitor. Tumor tissues collected 2 h after treatment with 3D185 (n = 6 mice per group). Data are shown as the mean ± SD. *p < 0.05; **p < 0.01; ***p < 0.001 vs the vehicle group, determined by one-way ANOVA with Dunnett’s multiple-comparison test. G, Percentage of the stained areas of CD31 in MC38 tumor. Positive areas per field are expressed as means ± SD. *p < 0.05; **p < 0.01; ***p < 0.001, compared with vehicle group. H, Flow cytometric analysis of the MDSC subpopulation among CD45^+^ cells in subcutaneous MC38 tumors treated with vehicle or the indicated inhibitor (n = 6 mice per group). Data are presented as the mean ± SD. Significant differences compared to the vehicle group were determined using one-way ANOVA with Dunnett’s multiple-comparison test (*p < 0.05; **p < 0.01; ***p < 0.001).

**References**

1. Ai J, Chen Y, Peng X, Ji Y, Xi Y, Shen Y, Yang X, Su Y, Sun Y, Gao Y, et al: **Preclinical Evaluation of SCC244 (Glumetinib), a Novel, Potent, and Highly Selective Inhibitor of c-Met in MET-dependent Cancer Models.** *Mol Cancer Ther* 2018, **17:**751-762.

2. Kobayashi-Watanabe N, Sato A, Watanabe T, Abe T, Nakashima C, Sueoka E, Kimura S, Sueoka-Aragane N: **Functional analysis of Discoidin domain receptor 2 mutation and expression in squamous cell lung cancer.** *Lung Cancer* 2017, **110:**35-41.

3. Helsten T, Elkin S, Arthur E, Tomson BN, Carter J, Kurzrock R: **The FGFR Landscape in Cancer: Analysis of 4,853 Tumors by Next-Generation Sequencing.** *Clin Cancer Res* 2016, **22:**259-267.

4. Kubo T, Yamamoto H, Lockwood WW, Valencia I, Soh J, Peyton M, Jida M, Otani H, Fujii T, Ouchida M, et al: **MET gene amplification or EGFR mutation activate MET in lung cancers untreated with EGFR tyrosine kinase inhibitors.** *Int J Cancer* 2009, **124:**1778-1784.

5. Muller PA, Vousden KH: **p53 mutations in cancer.** *Nat Cell Biol* 2013, **15:**2-8.

6. Jia LQ, Osada M, Ishioka C, Gamo M, Ikawa S, Suzuki T, Shimodaira H, Niitani T, Kudo T, Akiyama M, et al: **Screening the p53 status of human cell lines using a yeast functional assay.** *Mol Carcinog* 1997, **19:**243-253.

7. Modi S, Kubo A, Oie H, Coxon AB, Rehmatulla A, Kaye FJ: **Protein expression of the RB-related gene family and SV40 large T antigen in mesothelioma and lung cancer.** *Oncogene* 2000, **19:**4632-4639.

8. Guagnano V, Kauffmann A, Wohrle S, Stamm C, Ito M, Barys L, Pornon A, Yao Y, Li F, Zhang Y, et al: **FGFR genetic alterations predict for sensitivity to NVP-BGJ398, a selective pan-FGFR inhibitor.** *Cancer Discov* 2012, **2:**1118-1133.

9. Liu H, Ai J, Shen A, Chen Y, Wang X, Peng X, Chen H, Shen Y, Huang M, Ding J, Geng M: **c-Myc Alteration Determines the Therapeutic Response to FGFR Inhibitors.** *Clin Cancer Res* 2017, **23:**974-984.

10. Zhang YW, Staal B, Essenburg C, Su Y, Kang L, West R, Kaufman D, Dekoning T, Eagleson B, Buchanan SG, Vande Woude GF: **MET kinase inhibitor SGX523 synergizes with epidermal growth factor receptor inhibitor erlotinib in a hepatocyte growth factor-dependent fashion to suppress carcinoma growth.** *Cancer Res* 2010, **70:**6880-6890.

11. Okamoto W, Okamoto I, Arao T, Kuwata K, Hatashita E, Yamaguchi H, Sakai K, Yanagihara K, Nishio K, Nakagawa K: **Antitumor action of the MET tyrosine kinase inhibitor crizotinib (PF-02341066) in gastric cancer positive for MET amplification.** *Mol Cancer Ther* 2012, **11:**1557-1564.

12. Doebele RC, Pilling AB, Aisner DL, Kutateladze TG, Le AT, Weickhardt AJ, Kondo KL, Linderman DJ, Heasley LE, Franklin WA, et al: **Mechanisms of resistance to crizotinib in patients with ALK gene rearranged non-small cell lung cancer.** *Clin Cancer Res* 2012, **18:**1472-1482.

13. Zou HY, Friboulet L, Kodack DP, Engstrom LD, Li Q, West M, Tang RW, Wang H, Tsaparikos K, Wang J, et al: **PF-06463922, an ALK/ROS1 Inhibitor, Overcomes Resistance to First and Second Generation ALK Inhibitors in Preclinical Models.** *Cancer Cell* 2015, **28:**70-81.

14. Song A, Kim TM, Kim DW, Kim S, Keam B, Lee SH, Heo DS: **Molecular Changes Associated with Acquired Resistance to Crizotinib in ROS1-Rearranged Non-Small Cell Lung Cancer.** *Clin Cancer Res* 2015, **21:**2379-2387.

15. Xu X, De Angelis C, Burke KA, Nardone A, Hu H, Qin L, Veeraraghavan J, Sethunath V, Heiser LM, Wang N, et al: **HER2 Reactivation through Acquisition of the HER2 L755S Mutation as a Mechanism of Acquired Resistance to HER2-targeted Therapy in HER2(+) Breast Cancer.** *Clin Cancer Res* 2017, **23:**5123-5134.
